# Accurate Breakpoint Mapping in Apparently Balanced Translocation Families with Discordant Phenotypes Using Whole Genome Mate-Pair Sequencing

**DOI:** 10.1371/journal.pone.0169935

**Published:** 2017-01-10

**Authors:** Constantia Aristidou, Costas Koufaris, Athina Theodosiou, Mads Bak, Mana M. Mehrjouy, Farkhondeh Behjati, George Tanteles, Violetta Christophidou-Anastasiadou, Niels Tommerup, Carolina Sismani

**Affiliations:** 1 Department of Cytogenetics and Genomics, The Cyprus Institute of Neurology and Genetics, Nicosia, Cyprus; 2 The Cyprus School of Molecular Medicine, The Cyprus Institute of Neurology and Genetics, Nicosia, Cyprus; 3 Wilhelm Johannsen Centre for Functional Genome Research, Department of Cellular and Molecular Medicine, University of Copenhagen, Copenhagen N., Denmark; 4 Cytogenetics Unit, University of Social Welfare and Rehabilitation Sciences, Genetics Research Center, Tehran, Iran; 5 Department of Clinical Genetics, The Cyprus Institute of Neurology and Genetics, Nicosia, Cyprus; 6 Department of Clinical Genetics, The Cyprus Institute of Neurology and Genetics and Archbishop Makarios III Medical Centre, Nicosia, Cyprus; Queen Mary Hospital, HONG KONG

## Abstract

Familial apparently balanced translocations (ABTs) segregating with discordant phenotypes are extremely challenging for interpretation and counseling due to the scarcity of publications and lack of routine techniques for quick investigation. Recently, next generation sequencing has emerged as an efficacious methodology for precise detection of translocation breakpoints. However, studies so far have mainly focused on *de novo* translocations. The present study focuses specifically on familial cases in order to shed some light to this diagnostic dilemma. Whole-genome mate-pair sequencing (WG-MPS) was applied to map the breakpoints in nine two-way ABT carriers from four families. Translocation breakpoints and patient-specific structural variants were validated by Sanger sequencing and quantitative Real Time PCR, respectively. Identical sequencing patterns and breakpoints were identified in affected and non-affected members carrying the same translocations. *PTCD1*, *ATP5J2-PTCD1*, *CADPS2*, and *STPG1* were disrupted by the translocations in three families, rendering them initially as possible disease candidate genes. However, subsequent mutation screening and structural variant analysis did not reveal any pathogenic mutations or unique variants in the affected individuals that could explain the phenotypic differences between carriers of the same translocations. In conclusion, we suggest that NGS-based methods, such as WG-MPS, can be successfully used for detailed mapping of translocation breakpoints, which can also be used in routine clinical investigation of ABT cases. Unlike *de novo* translocations, no associations were determined here between familial two-way ABTs and the phenotype of the affected members, in which the presence of cryptic imbalances and complex chromosomal rearrangements has been excluded. Future whole-exome or whole-genome sequencing will potentially reveal unidentified mutations in the patients underlying the discordant phenotypes within each family. In addition, larger studies are needed to determine the exact percentage for phenotypic risk in families with ABTs.

## Introduction

Apparently balanced translocations (ABTs) involve the exchange of genomic regions between non-homologous chromosomes, without the gain or loss of genetic material. The majority of ABT carriers are phenotypically normal; however, an association with abnormal phenotypes, including intellectual disability (ID) and other congenital abnormalities, has been estimated in 6–9% of *de novo* cases [1,2]. For familial ABTs present in phenotypically normal individuals, it is generally considered that the risk of phenotypic abnormality in their balanced offspring is very low. Even though the risk is low, there are many cases reported where ABTs present in phenotypically normal carriers have been associated with abnormal phenotypes in related patients [3–6].

Several mechanisms have been proposed to explain how familial ABTs can be implicated in the development of this type of clinical discordancy. Work performed by our group using Fluorescence In Situ Hybridization (FISH) [5] and array-Comparative Genomic Hybridization (array-CGH) [7] showed that in a number of cases it could be explained by the presence of cryptic, submicroscopic imbalances or complex chromosomal rearrangements (CCRs). A second mechanism could be functional homozygosity due to gene disruption by the translocation on one allele and unmasking of a recessive gene mutation on the allele inherited from the other parent [8]. Familial ABTs can also cause discordant phenotypes by disruption of imprinted genes [9,10]. Another possible mechanism is “position effect” with variable expression of genes near the translocation or other identified rearrangement breakpoints [11,12]. Finally, reduced penetrance is a well-known observation in many dominant traits [13,14].

Several cytogenetic and molecular methods such as chromosomal G-banding, FISH [5], array-CGH [7,15,16], and array painting [17–19] have been applied throughout the years to characterize ABTs in individuals with an abnormal phenotype. However, molecular cytogenetic techniques have their individual limitations, and can often be technically challenging and time-consuming [20]. In addition, these methods cannot define the rearrangement at nucleotide level and may fail in identifying smaller chromosomal imbalances [20,21]. The exact characterization of translocation breakpoints and/or identification of any cryptic genomic imbalances and complex rearrangements in ABT carriers are crucial for identifying single disease candidate genes at or near the breakpoints, as well as understanding the molecular mechanisms underlying such events. This, in turn, will aid in the accurate correlation of the chromosomal rearrangement(s) with the clinical phenotype [22].

Recent studies have demonstrated that next generation sequencing (NGS) is a powerful tool for characterizing and mapping translocations at the nucleotide level. Targeted [20,23,24] and/or whole-genome NGS approaches [25–27], have so far been aimed at the investigation of *de novo* translocations. To date, there is a very limited bibliography regarding familial ABTs, which consequently leads to several diagnostic dilemmas about prenatal diagnosis and phenotypic risk.

In the current study, we present a whole-genome next generation mate-pair sequencing (WG-MPS) investigation to rapidly and accurately map translocation breakpoints in nine familial two-way ABT carriers from four families. All samples have been previously analyzed with G-banding, FISH, and array-CGH to exclude any submicroscopic imbalances. Our main aim was to characterize the translocations in each family to the base-pair (bp) level, identify disease candidate genes, and possibly reveal additional structural rearrangements that could explain the phenotypic differences between family members carrying the same translocation. This is, to the best of our knowledge, the largest study applying NGS methodologies for the investigation of familial ABT cases with discordant phenotypes. We demonstrate that WG-MPS, together with PCR and Sanger sequencing, allowed successful delineation of 18 translocation breakpoints to the nucleotide level, identification of four genes disrupted at the breakpoints, as well as investigation of the possible mechanisms underlying ABT generation. As previously suggested, we also support the use of NGS-based methods for the clinical investigation of ABT carriers.

## Materials and Methods

### Consent Form Collection, Study Participants, and Preliminary Analyses

This study was approved by the National bioethics committee as part of the Translation Facility Application with number EEBK/EΠ/2-13/09. Written informed consent was obtained from all participants, or adults responsible for children, before the beginning of this study.

Nine familial apparently balanced translocation (ABT) cases, from four different families, were included in this study. Each family had at least one clinically affected and one non-affected carrier of the same translocation.

Karyotype analysis using G-banding at the 550–750 band level on cultured lymphocyte metaphases, FISH using subtelomeric specific probes and/or whole chromosome paints [5], as well as array-CGH [7] with 1Mb or 200kb resolution chip were previously performed according to standard protocols.

### Mate-Pair Library Preparation and Sequencing

DNA samples were extracted from blood with the QIAamp DNA Blood Midi Kit (Qiagen, Hilden, Germany) according to the manufacturer’s recommendations. The concentration/quality of all DNA samples was quantified, prior to use, with NanoDrop. Mate-pair libraries were prepared by following the Nextera Mate-Pair Sample Preparation Guide, gel-free protocol (Part #15035209 Rev.C) obtained from Illumina’s webpage (http://www.illumina.com/) and using the Nextera Mate-Pair Sample Preparation kit (Illumina, San Diego, CA, USA).

In brief, 1 μg of genomic DNA was fragmented into 2-4kb inserts and tagged with a biotinylated mate-pair junction adaptor using a mate-pair tagment enzyme. The short single-stranded sequencing gaps left from the tagmentation reaction were filled using a strand displacement polymerase. The DNA fragments were then purified using Agencourt AMPure XP beads (Beckman Coulter Inc., Brea, CA, USA) followed by DNA fragment circularization and blunt-end ligation using a circularization ligase enzyme. The large circularized molecules were digested by sonication into smaller-sized fragments of ~200-400bp. Following that, biotinylated mate-pair DNA fragments were isolated by binding to streptavidin-coated beads (Dynabeads® M-280 Streptavidin—Invitrogen, Life Technologies, Carlsbad, CA, USA), and end-repaired to convert overhangs into blunt ends. A single ‘A’ nucleotide was added to the 3’ends of the fragments, which served to ligate Illumina TruSeq paired-end index adapters containing a complimentary ‘T’ nucleotide overhang. The mate-pair libraries were PCR amplified and the amplified products were purified with AMPure XP beads (Beckman Coulter Inc.). The final concentration of the mate-pair libraries was measured with Quant-iT™ PicoGreen® (Invitrogen) by following standard procedures. MPS libraries from each sample were diluted accordingly in water to 10nM and pooled into groups of four. Each pool was sequenced, as 100-bp paired-end reads, on a single flow-cell lane of the Illumina HiSeq2500 (Illumina) following the manufacturer’s protocol.

### Analysis of Mate-Pair Sequencing Data

Biotinylated adapter sequences were removed prior to alignment of high quality paired-end reads to the human reference genome GRCh37/hg19 using Burrows-Wheeler Aligner (BWA)-MEM with default parameters [28]. Reads not aligning uniquely as well as PCR duplicates were not analysed further. SVDetect was then used to identify potential translocations, inversions and large deletions/insertions from discordant paired-end data [29] aligning on different chromosomes, with unexpected strand orientation and at a distance deviating from the pre-defined insert length, respectively. These predicted structural variants (SVs) were also compared with in-house MPS data sets in order to identify SVs uniquely present in our familial ABT samples. Only SV predictions supported by at least five independent read-pairs were taken into consideration for further analysis. Visualization of the processed MPS data was performed using Integrative Genomics Viewer (IGV) (Broad Institute) [30] and the UCSC Genome Browser [31] (https://genome.ucsc.edu/).

### Translocation Breakpoints Validation

PCR primer pairs (Metabion, Planegg, Germany) were designed flanking the breakpoint junction of each reconstructed derivative chromosome using the Primer3 web interface tool [32] (http://bioinfo.ut.ee/primer3-0.4.0/) (S1 and S2 Figs, S1 Supporting Document). PCR amplification was performed in a 25μl reaction containing DNA sample, forward and reverse PCR primers (10μM) (Metabion), PCR buffer (1X) (Applied Biosystems, Life Technologies, Foster City, CA, USA), dNTPs (200μM) (Sigma-Aldrich, St. Louis, MO, USA), AmpliTaq polymerase (0.025μM) (Applied Biosystems), and dH_2_0. PCR primer sequences and conditions are listed in S1 Table. Amplified products were visualized on a 2% agarose gel, which was stained in ethidium bromide (0.5μg/ml) for 30 minutes. PCR products with single, clear bands were purified using ExoSAP-IT^®^ (Affymetrix, Santa Clara, CA, USA). Purified DNA (8ng/μl) was then cycle sequenced using forward or reverse primer (1μM) (Metabion), BigDye® Terminator v1.1 Ready Reaction Mix (Applied Biosystems), BigDye® Terminator Sequencing Buffer (1X) (Applied Biosystems), and deionized water in a 20μl reaction (cycle sequencing conditions available in S2 Table). Cycle sequencing cleanup was performed using Performa® DTR Gel Filtration Cartridges (EdgeBio, Gaithersburg, MD, USA), and purified sequencing reactions were run on a 3130xl Genetic Analyzer (Applied Biosystems). Translocation junction sequences were aligned to the reference genome using BLAT tool from the UCSC Genome Browser (https://genome.ucsc.edu/cgi-bin/hgBlat) [33].

### Translocation Generation and Position Effect Investigation

In order to get an insight into the mechanisms underlying translocation formation, the area spanning the translocation junctions was studied for the presence of any repetitive elements using the RepeatMasker track from the UCSC Genome Browser. In addition, a file containing highly conserved non-coding sequences (CNEs), found to be associated with developmental regulators [34], was uploaded as a custom track. This was done to investigate whether the breakpoints disrupted any of these conserved sequences, and in turn, possibly disrupted the regulation of important developmental genes located within the same topological domain [35] as the CNEs (long-range position effect).

### Unmasking of a Recessive Mutation Investigation

Intronic PCR primers (Metabion) flanking the exons and intron-exon boundaries of all disrupted genes were designed using Primer3 (PCR primer sequences and conditions are available in S3 Table). Before ordering, primer specificity was checked using the *in-silico* PCR tool from the UCSC Genome Browser. Bi-directional mutation screening on the alternative allele was performed using the BigDye® Terminator v1.1 Cycle Sequencing kit (Applied Biosystems) followed by analysis on a 3130xl Genetic Analyzer (Applied Biosystems). Cycle sequencing reactions and conditions were the same as in the validation of translocation breakpoints (S2 Table). Variant detection was done using the BLAT tool from the UCSC Genome Browser (https://genome.ucsc.edu/cgi-bin/hgBlat) [33].

### Uniparental Disomy (UPD) Investigation

Genotyping for UPD7 [36] in family 1 was done by mixing DNA sample from each member with a mixture of eight fluorescent chromosome (chr)7-specific repeat microsatellite markers (listed in S4 Table) and a multiplex PCR master mix (Qiagen). Following PCR amplification, amplified DNA from each sample was mixed with Hi-Di™ Formamide and GeneScan™–500 LIZ® Size Standard DNA ladder (Applied Biosystems). Finally the samples were run using a Fragment Analysis protocol on a 3130xl Genetic Analyzer (Applied Biosystems) according to the manufacturer’s instructions. UPD7 data analysis was done using the Applied Biosystem GeneMapper v4.1 Software.

### Structural Variant Analysis

Processed MPS data were filtered further (detailed description in S1 Supporting Document) in order to exclude variants overlapping by ≥80% with common SVs reported in the Database of Genomic Variants (DGV) [37], and identify patient-specific SVs within each family that could contribute to the observed discordant phenotypes.

### Quantitative Real-Time PCR (qRT-PCR) Analysis

In order to validate selected patient-specific structural variants, qRT-PCR was performed using SsoFast™ Evagreen® Supermix (Bio-Rad Laboratories Inc., Hercules, CA, USA) and Primer3-designed PCR primer pairs (0.5μM) (Metabion) spanning the SVs (qRT-PCR primer sequences are available in S5 Table). An internal control primer-pair at 18q12.2 was selected to confirm the efficiency of the reaction and normalize the results. All reactions for each primer pair were run in triplicates on a CFX96 Touch™ Real-Time PCR Detection System machine (Bio-Rad Laboratories, Inc.), and results were analyzed using the Bio-Rad CFX Manager analysis software (comparative Ct method).

### Reference Sequences

The GRCh37/hg19 reference genome assembly was used for annotation of the WG-MPS data, as well as primer design and data analysis for Sanger sequencing, mutation screening on the alternative allele of the disrupted genes and qRT-PCR. Annotation of variants identified after mutation screening was done according to GenBank reference sequences: NM_017954.10 for *CADPS2* and NM_001199013.1 for *STPG1*.

## Results

All nine familial ABT cases were previously found to have apparently balanced translocations using G-banding, FISH, and array-CGH at the resolution of 1Mb or 200kb. As these families still remained undiagnosed, a WG-MPS approach was employed as a next step in order to define the breakpoints to the base-pair level, detect additional SVs and make genotype-phenotype correlations. Results of the individual families are presented below:

### Family 1

**Fig 1:**
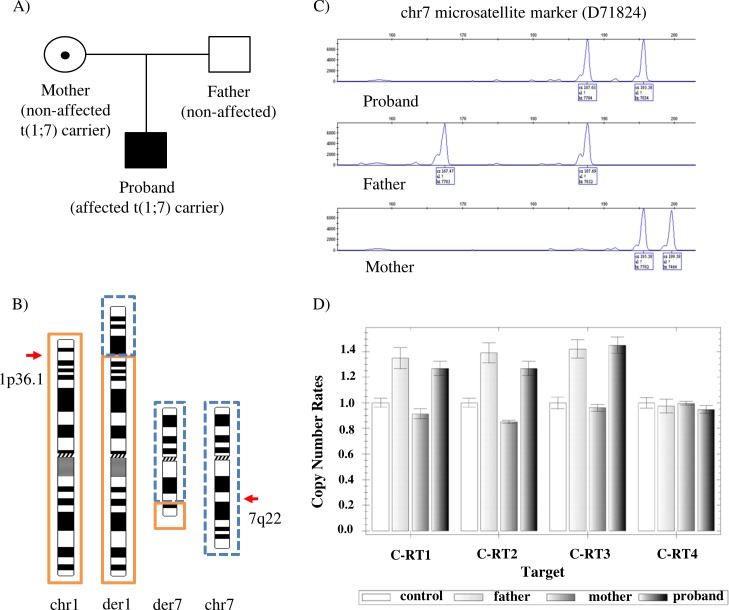
Family 1 results with t(1;7) translocation. A) Family 1 pedigree showing the proband with intellectual disability, psychomotor delay and epilepsy, as well as the non-affected mother and father. The t(1;7)(p36.1;q22) translocation is maternally inherited. B) Ideograms showing the normal and derivative (der) chromosomes (chr) 1 and 7 (not to scale). Genetic material from chr1 and chr7 is shown with a solid, orange and dotted, blue frame line, respectively. The approximate breakpoint positions on 1p36.1 and 7q22 are indicated by arrows. C) UPD7 analysis results from one of the informative microsatellite markers (D71824) in the affected proband and non-affected parents; by comparing the peak sizes between all family members, normal biparental inheritance was concluded. D) Quantitative Real-Time PCR results demonstrating the paternal inheritance of the chr3 duplication predicted from the structural variant analysis in the affected proband.

**Fig 2:**
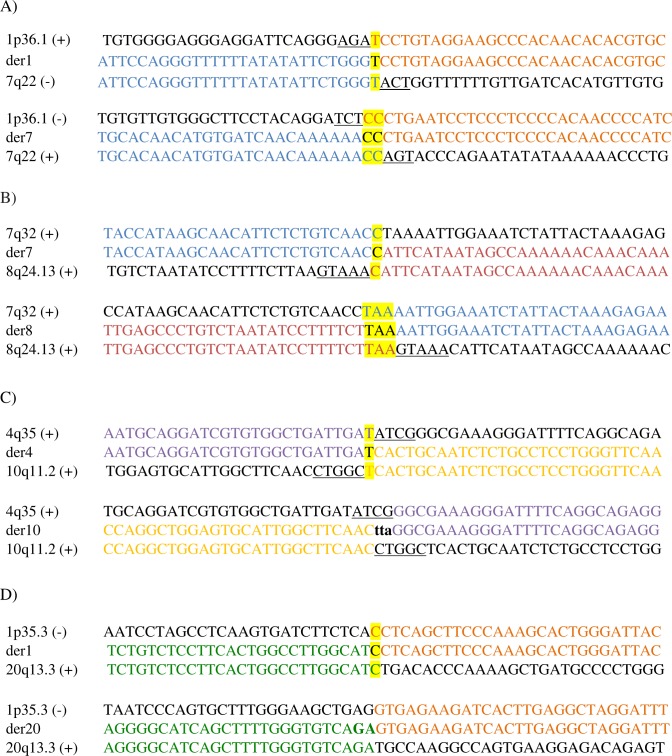
Translocation junction sequences identified in each family. Translocation junction sequences to the base-pair level as identified by mate-pair sequencing and verified by Sanger sequencing in A) family 1 with t(1;7)(p36.1;q22), B) family 2 with t(7;8)(q32;q24.13), C) family 3 with t(4;10)(q35;q11.2), and D) family 4 with t(1;20)(p35.3;q13.3). Translocation junction sequences (middle line) and matching reference sequences (top and bottom lines) are shown with a different colour depending on the chromosome involved (chr1-orange; chr4-purple; chr7-blue; chr8-red; chr10-yellow; chr20-green). Microhomology observed at the translocation breakpoint sites is highlighted in yellow, deleted sequences around the breakpoints are underlined, duplicated sequences are in bold, capital letters, and inserted sequences not aligning to either chromosome are in bold, lower-case letters.

At the time of the study, the patient was a 14-year old male with ID, psychomotor delay and epilepsy, carrying a t(1;7)(p36.1;q22) translocation inherited from his non-affected mother (Fig 1A and 1B). Translocation junction coordinates as predicted by MPS in the affected male and his non-affected mother are included in Table 1. Sequencing patterns and translocation breakpoint positions were identical in both family members. Repetitive elements, small imbalances, and microhomology sequences were also observed at each breakpoint site (Fig 2A; Table 1). The breakpoint on der(1) did not disrupt any known gene or CNE located around developmental regulatory genes. The breakpoint on der(7) disrupted *PTCD1* (Pentatricopeptide repeat domain 1) (OMIM 614774) (intron 7), as well as the *ATP5J2-PTCD1* read-through transcript (intron 8) (Table 1). Sequencing of all protein-coding exons in the affected member did not detect any sequence variation as compared to the reference sequence (GRCh37/hg19). In addition, UPD of chromosome 7 was excluded (Fig 1C; S4 Table).

**Table 1:** Breakpoint mapping and sequencing results for each apparently balanced translocation case included in this study.

Case / phenotype	Translocation junction as estimated by MPS	Junction length	Read-pairs	Translocation breakpoint position as defined by SS	Disrupted gene(s)	Indels (+strand)	Microho- mology	Repetitive elements
**Family 1 –t(1;7)(p36.1;q22)**								
Male with ID, psychomotor delay,epilepsy	chr1:18163342–18163563	222bp	12	chr1:18163344–18163348	—	3bp-AGT del.	T	SINE-MIR-MIRb
	chr7:99019714–99019855	142bp		chr7:99019710–99019714	*PTCD1* & *ATP5J2-PTCD1*	3bp-AGA del.	CC	LINE-L1-L1M5
Non-affected mother	chr1:18163366–18163436	71bp	17	chr1:18163344–18163348	—	3bp-AGT del.	T	SINE-MIR-MIRb
	chr7:99019342–99019746	405bp		chr7:99019710–99019714	*PTCD1* & *ATP5J2-PTCD1*	3bp-AGA del.	CC	LINE-L1-L1M5
**Family 2 –t(7;8)(q32;q24.13)**								
Female with ID	chr7:122515289–122515690	402bp	17	chr7:122515671–122515672	*CADPS2*	—	C	—
	chr8:119865523–119866376	854bp		chr8:119866044–119866050	—	5bp-GTAAA del.	TAA	—
Non-affected sibling	chr7:122514386–122515726	1341bp	20	chr7:122515671–122515672	*CADPS2*	—	C	—
	chr8:119866031–119866086	56bp		chr8:119866044–119866050	—	5bp-GTAAA del.	TAA	—
**Family 3 –t(4;10)(q35;q11.2)**								
Female with mild to moderate ID	chr4:189742584–189742790	207bp	15	chr4:189742651–189742656	—	4bp-ATCG del.	T	LINE-L2-L2a
	chr10:43139092–43140045	954bp		chr10:43139266–43139272	—	5bp-CTGGC del.	—	SINE-Alu-AluSc
Non-affected sibling	chr4:189742123–189743225	1103bp	26	chr4:189742651–189742656	—	4bp-ATCG del.	T	LINE-L2-L2a
	chr10:43139186–43139369	184bp		chr10:43139266–43139272	—	5bp-CTGGC del.	—	SINE-Alu-AluSc
Non-affected mother	chr4:189742483–189743387	905bp	25	chr4:189742651–189742656	—	4bp-ATCG del.	T	LINE-L2-L2a
	chr10:43139065–43140359	1295bp		chr10:43139266–43139272	—	5bp-CTGGC del.	—	SINE-Alu-AluSc
**Family 4 –t(1;20)(p35.3;q13.3)**								
Male with Polysynda-ctyly, Oral Anomalies	chr1:24738004–24738807	804bp	13	chr1:24738180–24738181	*STPG1*	—	C	SINE-Alu-AluJr4
	chr20:56177192–56177656	465bp		chr20:56177612–56177613	—	2bp-GA dupl.	—	—
Non-affected mother	chr1:24738108–24738220	113bp	15	chr1:24738180–24738181	*STPG1*	—	C	SINE-Alu-AluJr4
	chr20:56177454–56178424	971bp		chr20:56177612–56177613	—	2bp-GA dupl.	—	—

Translocation junction and exact breakpoint position as identified by Mate Pair Sequencing (MPS) and Sanger sequencing (SS), respectively, in the affected and non-affected translocation carriers in each family. The number of read-pairs representing each translocation junction, the gene(s) disrupted by each translocation breakpoint as well as insertions/deletions (indels), microhomology and repetitive elements found at the breakpoint sites are indicated. All genomic coordinates are based on the GRCh37/hg19 reference genome assembly. (ID = Intellectual Disability; mat = maternal; bp = base-pair; del. = deletion; dupl. = duplication)

After filtering our MPS data for common SVs reported previously in DGV (S1 Supporting Document), nineteen patient-specific SVs (represented by ≥5 reads) were identified (S6 Table). A large duplication (chr3:50382561–50403806) supported by 22 read-pairs, was selected to be investigated further with qRT-PCR using DNA samples from the proband, parents and unrelated control, and four PCR primer-pairs spanning the predicted duplicated area. Results revealed a relative copy number increase in both affected proband and non-affected father in the case of C-RT1, C-RT2, and C-RT3 primer-pairs, while normal results were observed across all samples relative to the control in the case of C-RT4 primer-pair (Fig 1D).

### Family 2

In the second family, two female siblings are carriers of a t(7;8)(q32;q24.13) translocation. The first sibling was diagnosed with ID, while the other was phenotypically normal. Table 1 includes the MPS-predicted translocation breakpoint junctions on der(7) and der(8) as well as the identical breakpoint positions identified in both siblings (Table 1). Based on the sequencing results, none of the breakpoints were located within repetitive sequences. However, 1bp (C) and 3bp (TAA) microhomology was present at each breakpoint site (Fig 2B). The der(8) breakpoint did not disrupt any protein-coding genes or CNEs. The der(7) breakpoint caused the disruption of *CADPS2* (calcium-dependent secretion activator 2) (OMIM 609978) (intron 1) (Table 1). Sequencing of all *CADPS2* exons on the alternative allele in the affected member detected a single known synonymous polymorphic variant (rs2251761) on exon 3 (NM_017954.10) (S3 Fig).

**Fig 3:**
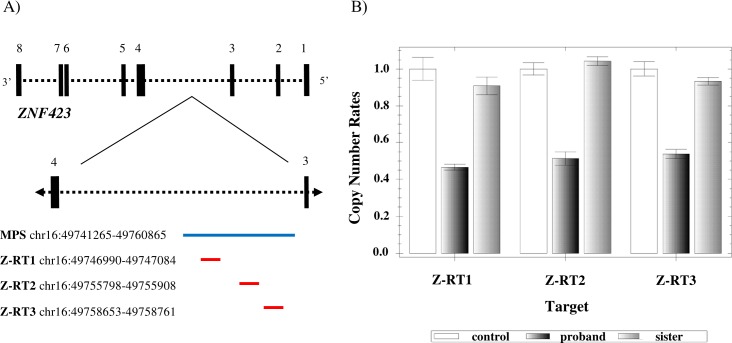
The *ZNF423* gene and quantitative Real-Time PCR (qRT-PCR) results. A) Schematic illustration of the *ZNF423* gene (minus strand). The protein-coding exons and introns of the gene are represented with solid, vertical lines and dotted, horizontal lines, respectively (not to scale). Intron 3 is enlarged to demonstrate the approximate *ZNF423* deletion position (blue line) as predicted by MPS in the affected sibling in family 2, as well as the amplicon (Z-RT1, Z-RT2, and Z-RT3) positions (red lines) from the qRT-PCR analysis. The actual coordinates are given on the left (GRCh37/hg19). B) qRT-PCR results demonstrating a reduced relative *ZNF423* copy number in the proband and normal results in the non-affected sibling as compared with a control. Parental genomic material was unavailable and thus not included in the analysis.

Six patient-specific SVs were identified (S7 Table) and only two of them disrupted known genes, namely *SH3RF3* (SH3 domain containing ring finger 3) and *ZNF423* (zinc finger protein 423) (OMIM 604557). The ~19.6kb deletion (chr16:49741265–49760865) supported by 17 read-pairs and disrupting *ZNF423* was investigated further with qRT-PCR using DNA samples from the affected proband, non-affected sibling and control, as well as three primer-pairs (Z-RT1, Z-RT2, and Z-RT3) spanning the predicted deletion (Fig 3A). Parental genomic material was unavailable and thus not included in the analysis. A relative *ZNF423* copy number decrease was observed in the affected proband, but not in the non-affected sibling, compared with a control sample (Fig 3B). Sequencing of all *ZNF423* exons and exon-intron boundaries revealed no sequence variations as compared to the reference sequence (GRCh37/hg19).

### Family 3

The affected member in family 3 was, at the time of the study, a 42-year old female presenting with mild to moderate ID. Chromosome analysis revealed a t(4;10)(q35;q11.2) translocation which was shared between her non-affected brother and non-affected mother. Individual translocation breakpoint junctions as well as identical breakpoint positions and sequencing patterns in all family members are indicated in Table 1 and Fig 2C. No genes were disrupted by the translocation breakpoints. In addition, no CNEs were located up to 1Mb upstream or downstream either breakpoint.

Eleven different SVs, not recorded in DGV, were identified uniquely in the affected translocation carrier (S8 Table). Only 4 rearrangements disrupted known genes: *ATG4C* (autophagy related 4C, cysteine peptidase) (OMIM 611339), *RAP1GDS1* (RAP1, GTPase-GDP dissociation stimulator 1) (OMIM 179502), *TP53TG3* (TP53 target 3) and *ZNF383* (zinc finger protein 383). Based on their functions none appeared to be associated with intellectual disability presented in the patient, therefore, no further investigation was carried out.

### Family 4

At the time of the study, the patient in family 4 was a 3-year old male presenting with polysyndactyly and oral anomalies. Chromosome analysis revealed a t(1;20)(p35.3;q13.3) translocation which was inherited from his non-affected mother. Despite the different translocation junctions predicted by MPS, the breakpoint positions and sequencing patterns were exactly the same in both family members (Table 1). Microhomology (1bp-C) was observed only at the der(1) breakpoint, which was also located within SINE/Alu/AluJr4 repeats (Table 1; Fig 2D). Disruption of *STPG1* (Sperm-tail PG-rich repeat containing 1) (OMIM 615826) (intron 1) occurred at the der(1) breakpoint site, whereas no CNEs were located at either breakpoint region. Sequencing of all *STPG1* gene exons on the alternative allele in the patient revealed two known polymorphic variants on exon 5 (NM_001199013.1): rs1064842 and rs1142057 (S4 Fig).

MPS data filtering for known variants reported in DGV and common variants between the two family members revealed six patient-specific SVs (S9 Table). A single known gene (*ECHDC1*- ethylmalonyl-CoA decarboxylase 1) (OMIM 612136) was disrupted by two separate SVs; however, it appears unlikely that it is causally linked to the patient’s phenotype.

## Discussion

Currently, there is very limited literature regarding the genetic causality of familial ABTs in cases where one member is clinically affected and the other(s) is/are phenotypically normal. To our knowledge, only a single study has addressed similar challenges and has investigated only one family. Specifically, large-insert paired-end tag NGS was used to investigate a t(6;8) balanced translocation shared between an asymptomatic mother and her two affected children. A disease candidate gene was disrupted at one of the two translocation breakpoint sites but no additional mutations on the other allele were detected. It was concluded that further functional studies were needed to establish pathogenicity [38]. The present study is the largest one conducted so far, which thoroughly investigated all possible mechanisms leading to discordant phenotypes, in nine cases with identical ABTs in each of the four families.

Initially, the genes disrupted by the ABT breakpoints that could potentially constitute disease candidate genes were identified through WG-MPS and subsequently Sanger sequencing. These were *PTCD1* and *ATP5J2-PTCD1* in family 1, *CADSP2* in family 2, and *STPG1* in family 4. *PTCD1* is a gene implicated in the regulation of mitochondrial gene expression and the negative regulation of leucine tRNA levels in the mitochondria [39,40]. Mitochondrial dysfunction has been linked to epilepsy [41], which agrees with the phenotype of the family 1 affected case. However, so far *PTCD1* has not been associated with inherited diseases. Furthermore, the function of the *ATP5J2-PTCD1* read-through transcript is completely unknown. Currently there exists minimal information regarding the function of *STPG1*, the gene disrupted in family 4 examined here, besides a potential role in induced apoptosis pathways [42]. In this study, subsequent examination of *PTCD1*, *ATP5J2-PTCD1* and *STPG1* coding exons on the alternative allele did not identify any point mutations in the affected members that could result in complete loss-of-function of these genes.

*CADPS2*, the gene disrupted by the translocation in family 2, maps within the autism susceptibility locus 1 on 7q31-q33 and has been linked in a number of studies with ID and autism [43,44]. It has been shown that knockout of *CADPS2* in mouse models impairs cerebellar development and function [45]. Knockout mice also display autistic-like behaviours, and alternative splicing of *CADPS2* mRNA lacking exon 3 has been observed in some autistic patients [43]. In a recent study, it was reported that *CADPS2* expression is subject to a parent-of-origin effect, with only the maternal allele being expressed in blood and amygdala [44]. However, because parental genomic material was not available in family 2 we could not determine whether the translocation was inherited, and also UPD7 was not excluded. Even though *CADPS2* could be a possible disease candidate gene based on its function and previous studies, no pathogenic point mutations were identified on the alternative allele in the affected individual that could explain the phenotypic differences between the two siblings. As the same translocation was also present in the phenotypically normal sibling, this observation suggests that if *CADPS2* disruption contributes to the patient’s phenotype, it must be through incomplete penetrance, imprinting or other mechanisms.

As a next step, a structural variant analysis was performed by filtering WG-MPS data for common variants reported in DGV. This enabled us to reduce the WG-MPS data volume and identify patient-specific variants that could possibly account for the discordant phenotypes observed between carriers of the same translocation. Selected disease candidate variants were confirmed by qRT-PCR. For example, a family 1 patient-specific large duplication (~21kb) was investigated further as disruption of *CACNA2D2* (calcium voltage-gated channel auxiliary subunit alpha2delta 2) (OMIM 607082), one of the genes within the duplicated region, has been previously reported in patients with epilepsy and developmental delay [46,47]. A relative copy number increase was detected in the affected proband, as compared with a control, by using primer-pairs C-RT1, C-RT2, and C-RT3 (Fig 1D). A normal copy number state was observed by using C-RT4, which is probably due to the fact that the amplified area falls outside the actual duplication breakpoints. The same profile was also observed in the non-affected father, suggesting that the duplication was paternally inherited. Since the same duplication was detected in both affected and non-affected individuals, it appears that the phenotypic consequence of the duplicated *CACNA2D2* gene (exons 36–39) is most probably non-pathogenic. However, as in the case of *CADPS2*, incomplete penetrance should also be considered.

qRT-PCR was also performed to validate a ~19.6kb deletion (chr16:49741265–49760865) disrupting *ZNF423* in the affected t(7;8) carrier in family 2. Reduced relative *ZNF423* copy number was observed in the affected sibling, as compared with a control, thus confirming the deletion (Fig 3B). Normal results were identified in the non-affected sibling; however, we could not determine whether the deletion was inherited as parental genomic material was unavailable. The *ZNF423* gene encodes for a zinc finger transcription factor (ZFP423) that is involved in cerebellar development [48,49]. 16q12 microdeletions encompassing *ZNF423* as well as other genes have been reported in patients with various developmental phenotypes [50–53]. The *ZNF423* deletion reported here is the smallest and covers *ZNF423* intron 2. Based on the amplicon positions showing a reduced relative *ZNF423* copy number, the deletion breakpoints can be refined between 49746990 and 49758761 (Fig 3A); however, the actual breakpoints may extend beyond these positions. Since *ZNF423* is highly expressed in the brain and *ZNF423* disruption results in phenotypes resembling those seen in our patient, we tested next whether there were any pathogenic mutations unmasked on the other allele. Sequencing though of all *ZNF423* exons did not detect any sequence variations in the affected translocation carrier as compared to the reference sequence (GRCh37/hg19).

To summarize, based on the results from a thorough investigation of the mechanisms that could possibly underlie the phenotypic differences between carriers of the same translocations, unmasking of recessive gene mutations by the translocations has been excluded, and wherever applicable, the possibility of uniparental disomy (family 1). Furthermore, none of the identified rearrangement breakpoints occurred within obvious candidate regions for long-range position effects. In families 1, 3, and 4, presence of cryptic imbalances or complex chromosomal rearrangements that could be causative for the discordant phenotypes observed within each family has also been excluded. The *ZNF423* deletion detected in the affected individual in family 2 may have a causative link to the patient’s phenotype; however, possible inheritance of the deletion has to be excluded, and functional studies are required in order to verify possible haploinsufficiency mechanisms underlying such positive association.

WG-MPS with long-insert libraries used in the current study proved highly successful in identifying familial ABT breakpoints. In total, eighteen translocation breakpoint junctions (two from each of the nine cases) were mapped down to a region ranging between 56bp and 1.3kb. This allowed further delineation to the nucleotide level with the use of a single-primer pair flanking each derivative chromosome breakpoint, per family, and by following standard PCR and Sanger sequencing procedures. In each family, identical breakpoint positions and sequencing patterns were identified in both affected and non-affected members carrying the same translocation. Similar to our study, the effective application of whole-genome NGS-based methods in identifying familial balanced translocation breakpoints was demonstrated in a recent study by Liang et al., 2016 [54]. In their parental study, eight families were investigated that could potentially carry balanced translocations based on prenatal cases with unbalanced karyotypes. G-banding and FISH analyses detected three balanced translocations and two submicroscopic balanced translocations, respectively, while low coverage whole-genome paired-end sequencing (WG-PES) detected all five translocations. It was concluded that WG-PES may replace conventional cytogenetic methods for the clinical identification of balanced translocation carriers [54], and results from our study using a similar NGS-based method further support this.

Apart from breakpoint mapping, sequencing to the base-pair level can also offer insights into possible mechanisms involved in ABT generation. In our study, molecular characterization of the breakpoints showed that they occurred in repetitive sequences in three out of four families. However, absence of homologous regions on both chromosomes involved in each translocation argues against non-allelic homologous recombination (NAHR). Instead, the presence of microhomology and/or small imbalances around the breakpoint sites (Table 1; Fig 2) suggests that the translocations were likely generated by microhomology-mediated repair (MHMR) [55] or non-homologous end-joining (NHEJ) [56] of double-strand breaks. These results come in agreement with previous studies reporting that following double-strand breaks, NHEJ is the predominant repair mechanism leading to translocations rather than NAHR [57].

In conclusion, our data has demonstrated that WG-MPS is a highly powerful tool that allows rapid and accurate mapping of familial ABT breakpoints as well as identification of single genes disrupted at the breakpoints and detection of additional patient-specific variants. A number of studies have identified genes disrupted by *de novo* translocations that are plausibly linked to the patient’s phenotype. However, based on the results presented here, it appears that in the majority of familial two-way ABT cases, in which the presence of cryptic imbalances or complex chromosomal rearrangements has been excluded, translocations are unrelated to the phenotype, unlike *de novo* translocations. Future analysis of our ABT families with whole-exome or whole-genome sequencing can potentially reveal the presence of unidentified pathogenic mutations in the affected individuals and provide additional evidence that the phenotype in the patients occurred independent of the detected ABTs. In addition, the exact frequency of the phenotypic risk in ABT families can be clarified in the future through the examination of larger cohorts of familial ABT cases, which will be of great importance for genetic counseling.

## Supporting Information

S1 Supporting Document Contains information regarding PCR primer design for the amplification and sequencing of the putative translocation junctions, and a more detailed explanation of the filtering steps for the Structural Variant analysis. (DOC)

S1 Fig **Primer design in case of a cis-joining translocation involving chromosomes (chr) A and B.** The illustrated ideograms were selected randomly to be used as examples here. The hypothetical breakpoints are shown with a dashed line. ChrA genetic material is illustrated with green, whereas, chrB with red.
(TIF)

S2 Fig Primer design in case of a trans-joining translocation involving chromosomes (chr) C and D. The illustrated ideograms were selected randomly to be used as examples here. The hypothetical breakpoints are shown with a dashed line. ChrC genetic material is illustrated with orange, whereas, chrD with blue.
(TIF)

S3 Fig Electropherogram showing the homozygous synonymous polymorphic variant (rs2251761) identified in the affected sibling in family 2 after mutation screening on the alternative allele of *CADPS2*. The codon changes from CTG (= Leu) to CTA (= Leu).
(TIF)

S4 Fig **Electropherograms showing the heterozygous synonymous and missense polymorphic variants: A) rs1064842 and B) rs1142057, respectively, identified in the affected member in family 4 after mutation screening on the alternative allele of *STPG1***.
A) In the case of rs1064842, the codon changes from AAT (= Asn) to AAC (= Asn).
B) In the case of rs1142057, the codon changes from ATT (= Ile) to GTT (= Val).
(TIF)

S1 Table PCR primer sequences and PCR conditions used for the amplification of each derivative chromosome junction in each family. The same PCR primers and PCR conditions were used in both affected and non-affected members carrying the same translocation within each family. The same general PCR protocol was used for all cases; different annealing temperatures (marked with a single asterisk) and extension times (marked with a double asterisk) used in each case are indicated.
t = translocation; der. = derivative; chr. = chromosome; F = forward; R = reverse; Tm = melting temperature; min = minutes; sec = seconds.
(DOC)

S2 Table Thermal cycler conditions used for the sequencing of amplified translocation junction sequences as well as exons of genes disrupted by the translocations. (DOC)

S3 Table List of PCR primers used for the exon amplification and sequencing of the genes disrupted by the translocation breakpoints in each affected member. The same general PCR protocol was used as in the amplification of the translocation junction sequences (included in S1 Table). The annealing temperatures and extension times used for the exon amplification of each gene were, respectively, 60°C and 1min for *PTCD1*, 60°C and 1min for *ATP5J2- PTCD1*, 61°C and 1min for *CADPS2*, and 61°C and 1min for *STPG1*.
chr. = chromosome; F = forward; R = reverse; Tm = melting temperature; bp = base pairs.
(DOC)

S4 Table Uniparental Disomy 7 (UPD7) results for family 1. List of fluorescent chromosome 7-specific repeat microsatellite markers used for UPD analysis in the affected t(1;7)(p36.1;q22) translocation carrier in family 1, non-affected t(1;7)(p36.1;q22) translocation carrier mother and non-affected father. Peak sizes from each marker in each sample are indicated as well as whether the result was informative (I) or not (NI). Based on the informative results, normal biparental inheritance was concluded. Results from D7S1824 (marked with an asterisk) were used as an example in Fig 1C (main manuscript).
(DOC)

S5 Table qRT-PCR primer sequences used for the validation of selected structural variants. chr. = chromosome; F = forward; R = reverse; Tm = melting temperature; bp = base pairs.
(DOC)

S6 Table List of filtered structural variants (SVs) (≥5 reads), not overlapping with any Database of Genomic Variants entry, found uniquely in the affected member of family 1. (DOC)

S7 Table List of filtered structural variants (SVs) (≥5 reads), not overlapping with any Database of Genomic Variants entry, found uniquely in the affected member of family 2. (DOC)

S8 Table List of filtered structural variants (SVs) (≥5 reads), not overlapping with any Database of Genomic Variants entry, found uniquely in the affected member of family 3. (DOC)

S9 Table List of filtered structural variants (SVs) (≥5 reads), not overlapping with any Database of Genomic Variants entry, found uniquely in the affected member of family 4. (DOC)
