# Correction: Accurate Breakpoint Mapping in Apparently Balanced Translocation Families with Discordant Phenotypes Using Whole Genome Mate-Pair Sequencing

**DOI:** 10.1371/journal.pone.0174190

**Published:** 2017-03-15

**Authors:** The PLOS ONE Staff

The legend for Table 1 is incorrectly displayed in the third paragraph of the Results section. The publisher apologizes for the error.

The complete Table 1 legend is: Translocation junction and exact breakpoint position as identified by Mate Pair Sequencing (MPS) and Sanger sequencing (SS), respectively, in the affected and non-affected translocation carriers in each family. The number of read-pairs representing each translocation junction, the gene(s) disrupted by each translocation breakpoint as well as insertions/deletions (indels), microhomology and repetitive elements found at the breakpoint sites are indicated. All genomic coordinates are based on the GRCh37/hg19 reference genome assembly. (ID = Intellectual Disability; mat = maternal; bp = base-pair; del. = deletion; dupl. = duplication).

**Table 1:** Breakpoint mapping and sequencing results for each apparently balanced translocation case included in this study.

Case / phenotype	Translocation junction as estimated by MPS	Junction length	Read-pairs	Translocation breakpoint position as defined by SS	Disrupted gene(s)	Indels (+strand)	Microho- mology	Repetitive elements
**Family 1 –t(1;7)(p36.1;q22)**								
Male with ID, psychomotor delay,epilepsy	chr1:18163342–18163563	222bp	12	chr1:18163344–18163348	—	3bp-AGT del.	T	SINE-MIR-MIRb
	chr7:99019714–99019855	142bp		chr7:99019710–99019714	*PTCD1* & *ATP5J2-PTCD1*	3bp-AGA del.	CC	LINE-L1-L1M5
Non-affected mother	chr1:18163366–18163436	71bp	17	chr1:18163344–18163348	—	3bp-AGT del.	T	SINE-MIR-MIRb
	chr7:99019342–99019746	405bp		chr7:99019710–99019714	*PTCD1* & *ATP5J2-PTCD1*	3bp-AGA del.	CC	LINE-L1-L1M5
**Family 2 –t(7;8)(q32;q24.13)**								
Female with ID	chr7:122515289–122515690	402bp	17	chr7:122515671–122515672	*CADPS2*	—	C	—
	chr8:119865523–119866376	854bp		chr8:119866044–119866050	—	5bp-GTAAA del.	TAA	—
Non-affected sibling	chr7:122514386–122515726	1341bp	20	chr7:122515671–122515672	*CADPS2*	—	C	—
	chr8:119866031–119866086	56bp		chr8:119866044–119866050	—	5bp-GTAAA del.	TAA	—
**Family 3 –t(4;10)(q35;q11.2)**								
Female with mild to moderate ID	chr4:189742584–189742790	207bp	15	chr4:189742651–189742656	—	4bp-ATCG del.	T	LINE-L2-L2a
	chr10:43139092–43140045	954bp		chr10:43139266–43139272	—	5bp-CTGGC del.	—	SINE-Alu-AluSc
Non-affected sibling	chr4:189742123–189743225	1103bp	26	chr4:189742651–189742656	—	4bp-ATCG del.	T	LINE-L2-L2a
	chr10:43139186–43139369	184bp		chr10:43139266–43139272	—	5bp-CTGGC del.	—	SINE-Alu-AluSc
Non-affected mother	chr4:189742483–189743387	905bp	25	chr4:189742651–189742656	—	4bp-ATCG del.	T	LINE-L2-L2a
	chr10:43139065–43140359	1295bp		chr10:43139266–43139272	—	5bp-CTGGC del.	—	SINE-Alu-AluSc
**Family 4 –t(1;20)(p35.3;q13.3)**								
Male with Polysynda-ctyly, Oral Anomalies	chr1:24738004–24738807	804bp	13	chr1:24738180–24738181	*STPG1*	—	C	SINE-Alu-AluJr4
	chr20:56177192–56177656	465bp		chr20:56177612–56177613	—	2bp-GA dupl.	—	—
Non-affected mother	chr1:24738108–24738220	113bp	15	chr1:24738180–24738181	*STPG1*	—	C	SINE-Alu-AluJr4
	chr20:56177454–56178424	971bp		chr20:56177612–56177613	—	2bp-GA dupl.	—	—

Translocation junction and exact breakpoint position as identified by Mate Pair Sequencing (MPS) and Sanger sequencing (SS), respectively, in the affected and non-affected translocation carriers in each family. The number of read-pairs representing each translocation junction, the gene(s) disrupted by each translocation breakpoint as well as insertions/deletions (indels), microhomology and repetitive elements found at the breakpoint sites are indicated. All genomic coordinates are based on the GRCh37/hg19 reference genome assembly. (ID = Intellectual Disability; mat = maternal; bp = base-pair; del. = deletion; dupl. = duplication).
