# Supplementary material for: Accurate Breakpoint Mapping in Apparently Balanced Translocation Families with Discordant Phenotypes Using Whole Genome Mate-Pair Sequencing
Source: PLoS One. 2017 Jan 10;12(1):e0169935. doi: 10.1371/journal.pone.0169935 (PMC5225008; doi:10.1371/journal.pone.0169935)
Supplement: S1 Supporting Document — (DOC) [file pone.0169935.s001.doc]

**S1 Supporting Document for:**

**Accurate Breakpoint Mapping in Apparently Balanced Translocation Families with Discordant Phenotypes Using Whole Genome Mate-Pair Sequencing**

Constantia Aristidou, Costas Koufaris, Athina Theodosiou, Mads Bak, Mana M. Mehrjouy, Farkhondeh Behjati, George Tanteles, Violetta Anastasiadou, Niels Tommerup, Carolina Sismani

**PCR Primer Design for Translocation Junction Amplification and Sequencing**

Primer design was slightly different depending on the p/q-arm genetic material exchange and the joining type of the translocations. That is, whether there was exchange of genetic material between the two p-arms or between the two q-arms of two non-homologous chromosomes (*cis*-joining), or whether there was exchange of genetic material between the p and q arms of two non-homologous chromosomes (*trans*-joining). These definitions were suggested by Dong et al. (2014) [1].

S1 Fig illustrates primer design for the amplification of the putative translocation junctions in a *cis*-joining translocation involving chromosomes (chr) A and B. In order to amplify the translocation breakpoint junction on derivative chromosome A (derA), a forward (F) primer on the left side of the identified putative translocation breakpoint region on chrA (chrA/F; light green arrow) (S1-i Fig) and a reverse (R) primer on the right side of the identified breakpoint region on chrB (chrB/R; dark red arrow) (S1-ii Fig) were selected. chrA/F and chrB/R are illustrated as derA/F and derA/R, respectively, on derA (S1-iii Fig). In order to amplify the translocation breakpoint junction on the derivative chromosome B (derB), a forward primer on the left side of the putative breakpoint region on chrB (chrB/F; light red arrow) (S1-ii Fig) and a reverse primer on the right side of the breakpoint region on chrA (chrA/R; dark green arrow) (S1-i Fig) were selected. chrB/F and chrA/R are illustrated as derB/F and derB/R, respectively, on derB (S1-iv Fig).

S2 Fig illustrates primer design for the amplification of the putative translocation junctions in a *trans*-joining translocation involving chromosomes (chr) C and D. In order to amplify the translocation breakpoint junction on derivative chromosome C (derC), a forward primer on the right side of the identified breakpoint region on chrD (chrD/R; dark blue arrow) (S2-ii Fig) and a reverse primer on the right side of the putative breakpoint region on chrC (chrC/R; dark orange arrow) (S2-i Fig) were selected. chrC/R and chrD/R are illustrated as derC/F and derC/R, respectively, on derC (S2-iii Fig). In order to amplify the translocation breakpoint junction on derivative chromosome D (derD), a forward primer on the left side of the putative breakpoint region on chrD (chrD/F; light blue arrow) (S2-ii Fig) and a reverse primer on the left side of the identified breakpoint region on chrC (chrC/F; light orange arrow) (Fig S2-i) were selected. chrD/F and chrC/F are illustrated as derD/F and derD/R, respectively, on derD (S2-iv Fig).

**Structural Variant Analysis**

To begin with, common structural variants recorded in DGV were downloaded from the Database of Genomic Variants (DGV) server (<http://dgv.tcag.ca/dgv/app/home>) [2] as a tab delimited text file format (GRCh37_hg19_variants_2015-07-23.txt). The coordinates are based on the GRCh37/hg19 reference genome. In order to filter for coverage as well as common variants from DGV and from an in-house data set, established from samples analyzed previously with mate-pair sequencing at the Department of Cellular and Molecular Medicine, University of Copenhagen, a program in Perl programming language was developed. The DGV text file and a bed file containing Structural Variants (SVs) detected from whole-genome MPS for each sample were then taken as input.

Only SVs uniquely found in each of our study samples and supported by ≥5 reads were kept for further filtering. Following that, each SV record in the individual sample bed file was compared with each record study in the DGV text file for any overlapping regions. A threshold overlap of ≥80% with DGV records was set; this means that if a variant recorded in our MPS data overlapped by 80% or higher with an entry recorded in DGV, then it was considered as a common variant and excluded for further investigation.

All recorded translocations, insertions, inverted duplications or inverted deletions were not filtered with DGV. Filtering was only applied to duplications, deletions and inversions. The records were not just compared based on overlap but also based on their annotation; an annotated SV in DGV as duplication, gain or gain+loss was considered as a match with duplication. An annotated SV in DGV as deletion, loss or gain+loss was considered as deletion.

Bedtools intersect function [3] was used to find unique events, not overlapping with DGV records, in the affected individuals of each family as compared with the non-affected individuals within the same family. Next, for families 1, 2, and 3, these identified unique events were compared with the ddg2p.bed file ([https://decipher.sanger.ac.uk/about#downloads/data](https://decipher.sanger.ac.uk/about#downloads/data)). This file contains a curated list of genes, from the Developmental Disorders Genotype-to-Phenotype database (DDG2P), that have been reported to be associated with developmental disorders [4].

**Supporting References**

1. Dong Z, Jiang L, Yang C et al. A robust approach for blind detection of balanced chromosomal rearrangements with whole-genome low-coverage sequencing. Hum Mutat 2014: 35: 625-636.
2. MacDonald JR, Ziman R, Yuen RK, Feuk L, Scherer SW. The Database of Genomic Variants: a curated collection of structural variation in the human genome. Nucleic Acids Res 2014: 42: D986-92.
3. Quinlan AR, Hall IM. BEDTools: a flexible suite of utilities for comparing genomic features. Bioinformatics 2010: 26: 841-842.
4. Wright CF, Fitzgerald TW, Jones WD, et al. Genetic diagnosis of developmental disorders in the DDD study: a scalable analysis of genome-wide research data. Lancet 2015: 385: 1305-1314.
