# Supplementary figures and images for: Accurate Breakpoint Mapping in Apparently Balanced Translocation Families with Discordant Phenotypes Using Whole Genome Mate-Pair Sequencing
Source: PLoS One. 2017 Jan 10;12(1):e0169935. doi: 10.1371/journal.pone.0169935 (PMC5225008; doi:10.1371/journal.pone.0169935)

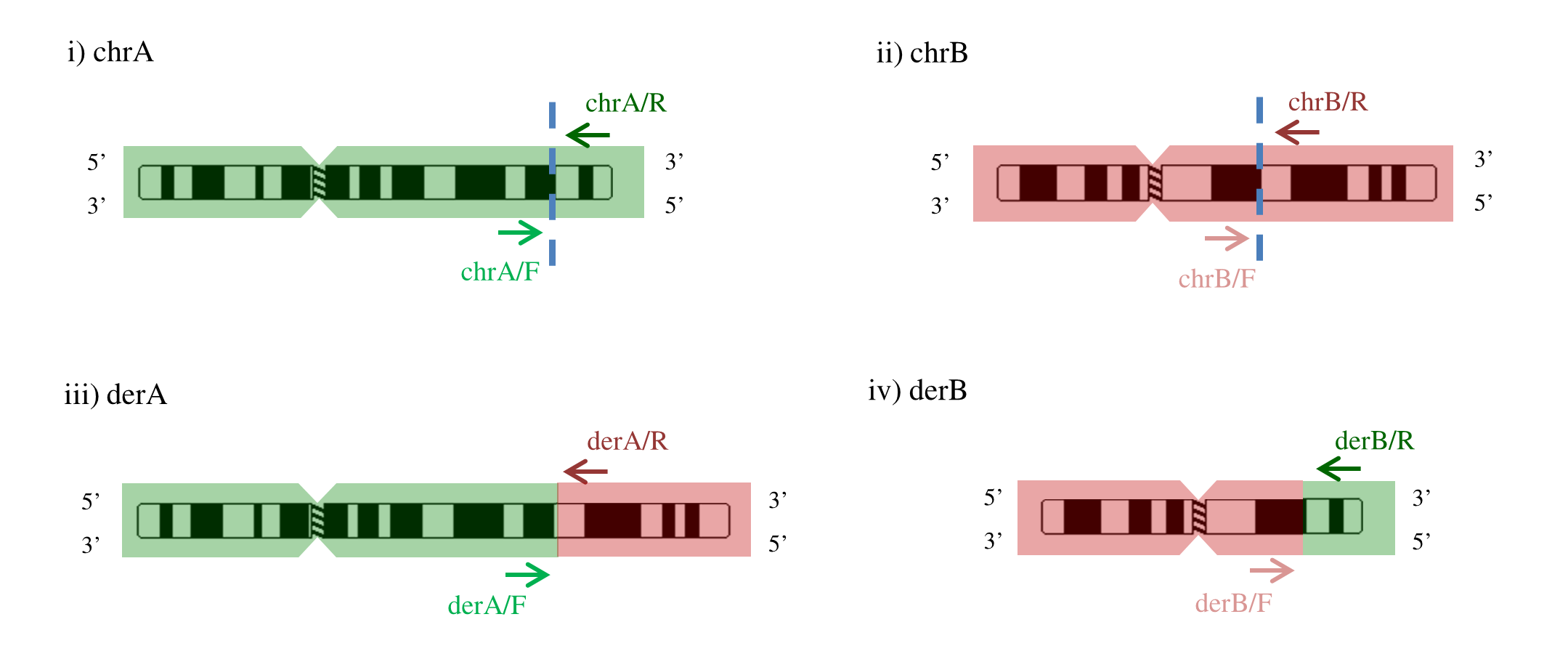

Supplement: S1 Fig — Primer design in case of a cis-joining translocation involving chromosomes (chr) A and B. The illustrated ideograms were selected randomly to be used as examples here. The hypothetical breakpoints are shown with a dashed line. ChrA genetic material is illustrated with green, whereas, chrB with red. (TIF) [file pone.0169935.s002.tif]

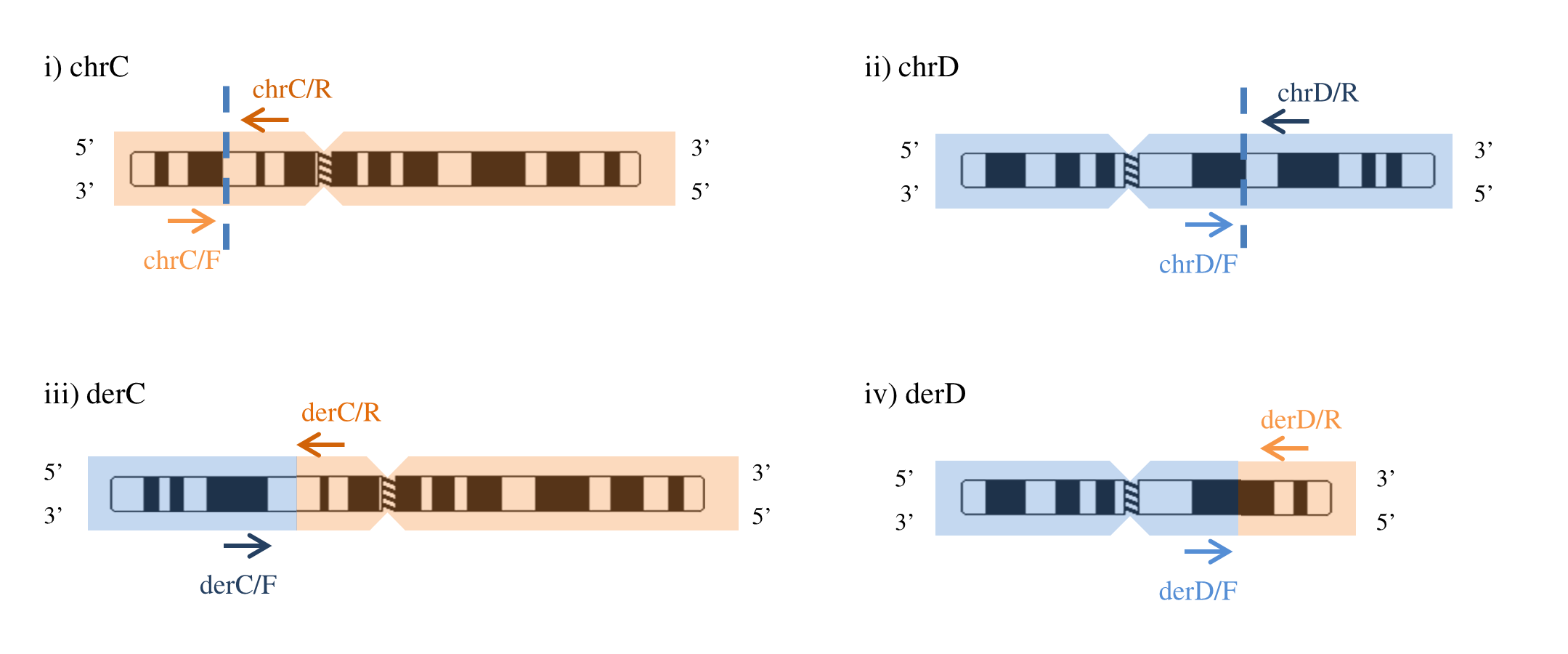

Supplement: S2 Fig — The illustrated ideograms were selected randomly to be used as examples here. The hypothetical breakpoints are shown with a dashed line. ChrC genetic material is illustrated with orange, whereas, chrD with blue. (TIF) [file pone.0169935.s003.tif]

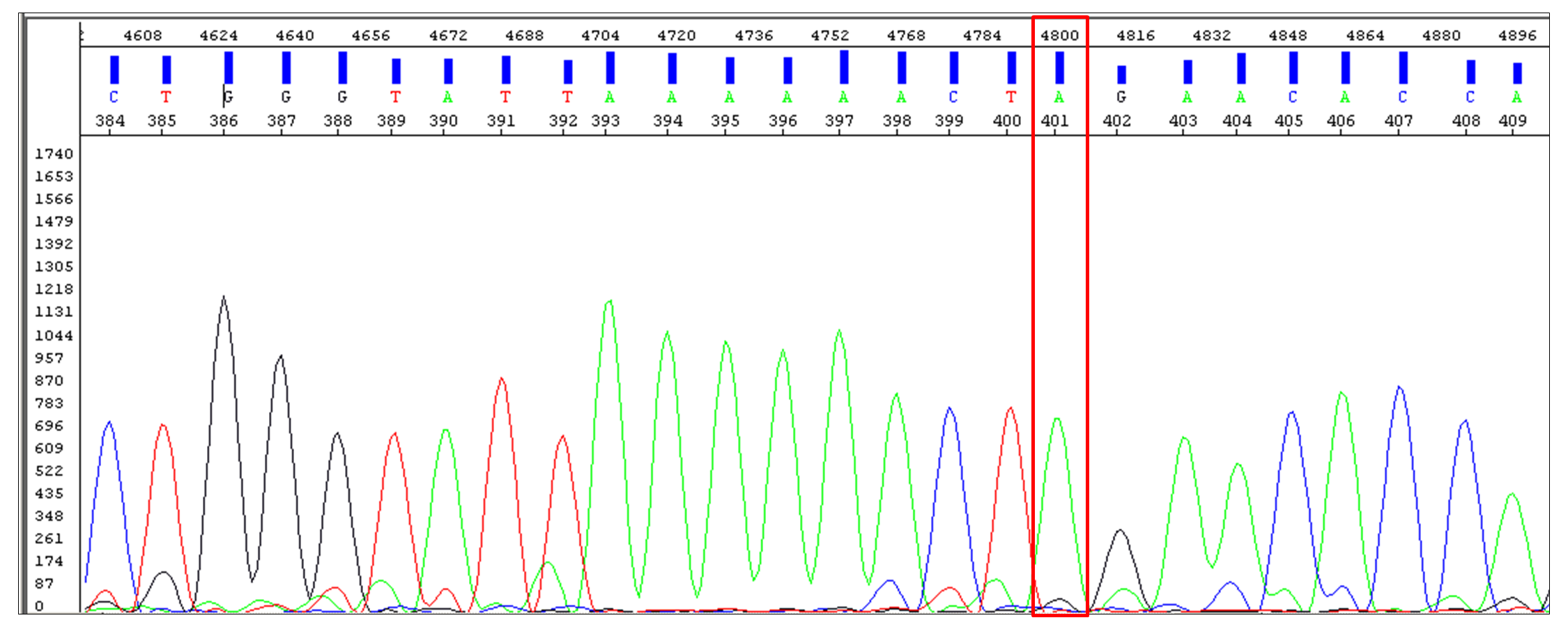

Supplement: S3 Fig — The codon changes from CTG (= Leu) to CTA (= Leu). (TIF) [file pone.0169935.s004.tif]

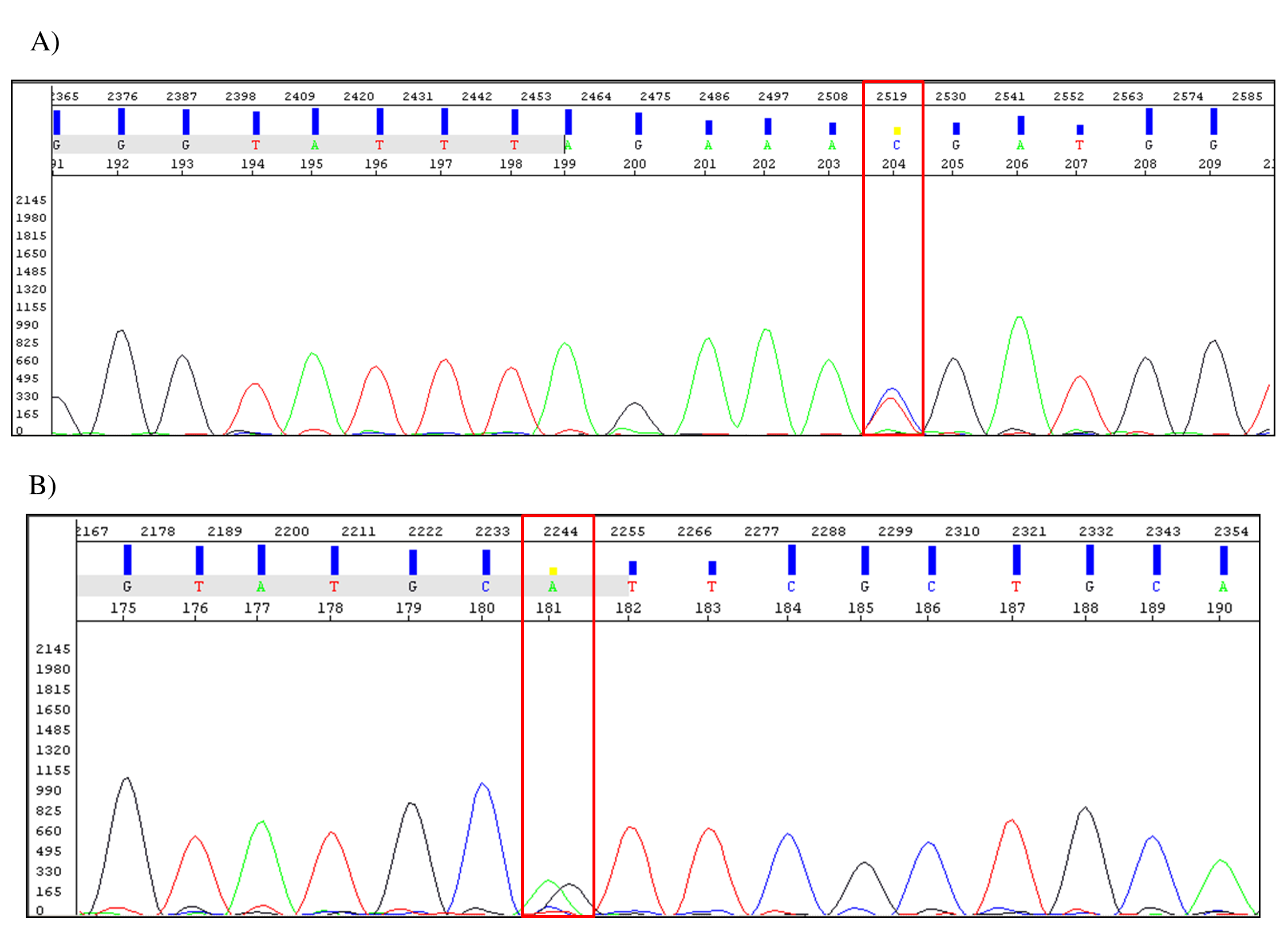

Supplement: S4 Fig — Electropherograms showing the heterozygous synonymous and missense polymorphic variants: A) rs1064842 and B) rs1142057, respectively, identified in the affected member in family 4 after mutation screening on the alternative allele of STPG1. A) In the case of rs1064842, the codon changes from AAT (= Asn) to AAC (= Asn). B) In the case of rs1142057, the codon changes from ATT (= Ile) to GTT (= Val). (TIF) [file pone.0169935.s005.tif]
