# Supplementary material for: Accurate Breakpoint Mapping in Apparently Balanced Translocation Families with Discordant Phenotypes Using Whole Genome Mate-Pair Sequencing
Source: PLoS One. 2017 Jan 10;12(1):e0169935. doi: 10.1371/journal.pone.0169935 (PMC5225008; doi:10.1371/journal.pone.0169935)
Supplement: S1 Table — The same PCR primers and PCR conditions were used in both affected and non-affected members carrying the same translocation within each family. The same general PCR protocol was used for all cases; different annealing temperatures (marked with a single asterisk) and extension times (marked with a double asterisk) used in each case are indicated. t = translocation; der. = derivative; chr. = chromosome; F = forward; R = reverse; Tm = melting temperature; min = minutes; sec = seconds. (DOC) [file pone.0169935.s006.doc]

**S1 Table. PCR primer sequences and PCR conditions used for the amplification of each derivative chromosome junction in each family.**

The same PCR primers and PCR conditions were used in both affected and non-affected members carrying the same translocation within each family. The same general PCR protocol was used for all cases; different annealing temperatures (marked with a single asterisk) and extension times (marked with a double asterisk) used in each case are indicated.

| **Family** | **Der. chr.** | **Primer name & sequence** | **Tm (^o^C)** | **General PCR protocol** | ***Annealing temperature** | ****Extension time** |
| --- | --- | --- | --- | --- | --- | --- |
| **Family 1 t(1;7)** | der1 | **der1F** GTAAATGGGCAGGGACACAC | 60.2 | **Step 1:** Initial denaturation  94 ^o^C for 3min  **Step 2:** Denaturation  94 ^o^C for 30sec  **Step 3:** Primer Annealing  * ^o^C for 30sec  **Step 4:** Polymerase Extension  72 ^o^C for **min  **Repeat steps 2-4: 32 more times**  **Step 5:** Final Extension  72 ^o^C for 3min  **Step 6**  4 ^o^C for ∞ | 60^o^C | 1min |
|  |  | **der1R** GCAGGACACTGTCACCAAAC | 59.2 |  |  |  |
|  | der7 | **der7F** AAGAAAACCTAGGCAACAAACTTC | 59.3 |  | 60^o^C | 1min |
|  |  | **der7R** CTTGCCCAAGTGTAGTCAGTG | 58.4 |  |  |  |
| **Family 2 t(7;8)** | der7 | **der7F** AAGAAAACCTAGGCAACAAACTTC | 59.3 |  | 60^o^C | 2min |
|  |  | **der7R** CTTGCCCAAGTGTAGTCAGTG | 58.4 |  |  |  |
|  | der8 | **der8F** CTGGCTGGCTTTGATGTCTA | 59.0 |  | 60^o^C | 2min |
|  |  | **der8R** TTTAGGATTACTTTATTTTTGGTTTTG | 57.7 |  |  |  |
| **Family 3 t(4;10)** | der4 | **der4F** CAAAGTAGGGTAATGGCATAGAAAA | 59.8 |  | 61^o^C | 2min |
|  |  | **der4R** CAAACTCTTCAGGGCATTGG | 60.6 |  |  |  |
|  | der10 | **der10F** CTCAAGAATCTGCCCACCTC | 59.8 |  | 61^o^C | 2min |
|  |  | **der10R** CACACCTAGAGCCCATCTCA | 58.8 |  |  |  |
| **Family 4 t(1;20)** | der1 | **der1F** ATGAGAGCCAAGGGAAAGTG | 59.3 |  | 60^o^C | 1min |
|  |  | **der1R** GAACTGTCCAACAGTGAACTTCC | 60.1 |  |  |  |
|  | der20 | **der20F** GCTTCAGCACTAAGGGGACA | 60.4 |  | 60^o^C | 1min |
|  |  | **der20R** CCGAGTGGGTATGATTGGTT | 59.7 |  |  |  |

t=translocation; der.=derivative; chr.=chromosome; F=forward; R=reverse; Tm=melting temperature; min=minutes; sec=seconds.
