# Supplementary material for: Accurate Breakpoint Mapping in Apparently Balanced Translocation Families with Discordant Phenotypes Using Whole Genome Mate-Pair Sequencing
Source: PLoS One. 2017 Jan 10;12(1):e0169935. doi: 10.1371/journal.pone.0169935 (PMC5225008; doi:10.1371/journal.pone.0169935)
Supplement: S2 Table — (DOC) [file pone.0169935.s007.doc]

**S2 Table. Thermal cycler conditions used for the sequencing of amplified translocation junction sequences as well as exons of genes disrupted by the translocations.**

| **Step** | **Temperature** | **Time** |  |
| --- | --- | --- | --- |
| 1 | 96 ^o^C | 1min |
| 2 | 96 ^o^C | 10sec | Repeat steps 2-4: 24 more times |
| 3 | 50 ^o^C | 5sec |
| 4 | 60 ^o^C | 4min |
| 5 | 4 ^o^C | ∞ |  |
