# Supplementary material for: Accurate Breakpoint Mapping in Apparently Balanced Translocation Families with Discordant Phenotypes Using Whole Genome Mate-Pair Sequencing
Source: PLoS One. 2017 Jan 10;12(1):e0169935. doi: 10.1371/journal.pone.0169935 (PMC5225008; doi:10.1371/journal.pone.0169935)
Supplement: S3 Table — The same general PCR protocol was used as in the amplification of the translocation junction sequences (included in S1 Table). The annealing temperatures and extension times used for the exon amplification of each gene were, respectively, 60°C and 1min for PTCD1, 60°C and 1min for ATP5J2- PTCD1, 61°C and 1min for CADPS2, and 61°C and 1min for STPG1. chr. = chromosome; F = forward; R = reverse; Tm = melting temperature; bp = base pairs. (DOC) [file pone.0169935.s008.doc]

**S3 Table. List of PCR primers used for the exon amplification and sequencing of the genes disrupted by the translocation breakpoints in each affected member.**

The same general PCR protocol was used as in the amplification of the translocation junction sequences (included in S1 Table). The annealing temperatures and extension times used for the exon amplification of each gene were, respectively, 60^o^C and 1min for *PTCD1*, 60^o^C and 1min for *ATP5J2- PTCD1*, 61^o^C and 1min for *CADPS2*, and 61^o^C and 1min for *STPG1*.

chr.=chromosome; F=forward; R=reverse; Tm=melting temperature; bp=base pairs.

| **Gene** | **Exon** | **Exon coordinates (hg19)** | **Primer name & sequence** | **Tm (^o^C)** | **Amplified region (hg19)** | **Amplicon size** |
| --- | --- | --- | --- | --- | --- | --- |
| *PTCD1* | 1 | chr7:99032413-99032891 | **PTCD1-1F**  AAGTGATATGCCAGCCTCGA | 58.9 | chr7:99032266-99033045 | 780bp |
|  |  |  | **PTCD1-1R**  GGGTTGCGATACTTACAGCC | 58.7 |  |  |
|  | 2 | chr7:99030901-99031041 | **PTCD1-2F**  ATGGTGTGAGGTGAGGGATG | 59.0 | chr7:99030727-99031215 | 489bp |
|  |  |  | **PTCD1-2R**  GGCTTTCCATGTCATCTCGTT | 58.6 |  |  |
|  | 3 | chr7:99027211-99027429 | **PTCD1-3F**  GTGTCTGAGTTTGGAGAGCTG | 58.6 | chr7:99027011-99027600 | 590bp |
|  |  |  | **PTCD1-3R**  AAAGCAGGACACAGCACAC | 58.6 |  |  |
|  | 4 | chr7:99026726-99026827 | **PTCD1-4F**  CTTTGTGCACTCGAGGGAAG | 58.9 | chr7:99026555-99027014 | 460bp |
|  |  |  | **PTCD1-4R**  CCTCTCCCTGCCATCTGTAG | 59.0 |  |  |

**S3 Table. Continued**

|  | 5 | chr7:99022418-99023239 |  |  |  |  |
| --- | --- | --- | --- | --- | --- | --- |
|  | 5.1 |  | **PTCD1-5.1F**  CGGTCTTGTCTCGAACTCCT | 59.5 | chr7:99022677-99023374 | 698bp |
|  |  |  | **PTCD1-5.1R**  AGTAGCGTGAGGGTCCTGAT | 58.8 |  |  |
|  | 5.2 |  | **PTCD1-5.2F**  CAGGCAACCTCATGTCAGC | 60.4 | chr7:99022255-99023043 | 789bp |
|  |  |  | **PTCD1-5.2R**  AAATGGGACCAAAGACCAGA | 59.4 |  |  |
|  | 6 | chr7:99021398-99021580 | **PTCD1-6F**  ATTGTTGCTGCCAAGTTTGC | 58.4 | chr7:99021268-99021712 | 445bp |
|  |  |  | **PTCD1-6R**  CTAAGGGAAGCTGCTGGACA | 59.4 |  |  |
|  | 7 | chr7:99017590-99017772 | **PTCD1-7F**  CTTTTCTGCCCTGTCCTCTG | 60.0 | chr7:99017516-99017911 | 396bp |
|  |  |  | **PTCD1-7R**  TGACACCAGCTCACTTGTCC | 59.9 |  |  |
|  |  |  |  |  |  |  |
| *ATP5J2-PTCD1* | 1 | chr7:99063752-99063824 | **ATP5J2-PTCD1-1F**  TCGCTTCATGGCCTTCTAGT | 60.0 | chr7:99063663-99063911 | 249bp |
|  |  |  | **ATP5J2-PTCD1-1R**  TTCTCTCCCAGTGTGGCTCT | 60.0 |  |  |
|  | 2 | chr7:99057709-99057816 | **ATP5J2-PTCD1-2F**  TGTGCAGCCTCTGTGTAATTG | 59.9 | chr7:99057571-99057942 | 372bp |
|  |  |  | **ATP5J2-PTCD1-2R**  ATCCCTCCCTGTCATCCTCT | 59.9 |  |  |

**S3 Table. Continued**

| *CADPS2* | 1 | chr7:122526053-122526813 |  |  |  |  |
| --- | --- | --- | --- | --- | --- | --- |
|  | 1.1 |  | **CADPS2-1.1F**  CGCTTACACGGGCCATCG | 61.3 | chr7:122526352-122526941 | 590bp |
|  |  |  | **CADPS2-1.1R**  CCTCGTCCGACTCCTCTTC | 59.0 |  |  |
|  | 1.2 |  | **CADPS2-1.2F**  TCCTGAATCTAACTCGACGTGT | 59.0 | chr7:122525900-122526552 | 653bp |
|  |  |  | **CADPS2-1.2R**  ACCGTCTTGTCCCCAAAGAT | 59.0 |  |  |
|  | 2 | chr7:122377009-122377122 | **CADPS2-2F**  GCCTGGAAGACAGAGCTTGA | 60.7 | chr7:122376827-122377325 | 499bp |
|  |  |  | **CADPS2-2R**  CCAGGCTGTGGAATTAGAGC | 59.9 |  |  |
|  | 3 | chr7:122303291-122303623 | **CADPS2-3F**  TCATTGTGGAGTATTTGGCTGAA | 58.3 | chr7:122303052-122303749 | 698bp |
|  |  |  | **CADPS2-3R**  AGGACACATGGGGATGATTACT | 58.6 |  |  |
|  | 4 | chr7:122269302-122269382 | **CADPS2-4F**  TTCTTGATTTGGTGGTTTTGTG | 59.9 | chr7:122269124-122269623 | 500bp |
|  |  |  | **CADPS2-4R**  GCTTCATTTAGATAACATGCTTTCAG | 59.8 |  |  |
|  | 5 | chr7:122261535-122261771 | **CADPS2-5F**  CAAAAATGCCAAAGCCTGA | 59.8 | chr7:122261386-122261939 | 554bp |
|  |  |  | **CADPS2-5R**  GCTGAGTGTCCAACAAATGG | 59.1 |  |  |
|  | 6 | chr7:122255235-122255353 | **CADPS2-6F**  CAGTTCATGCTGCTGTTTATGT | 57.6 | chr7:122255164-122255430 | 267bp |
|  |  |  | **CADPS2-6R**  CAGAGCATGTTTCTTTGGGAG | 57.1 |  |  |

**S3 Table. Continued**

|  | 7 | chr7:122221233-122221344 | **CADPS2-7F**  TGTGCCATCTGCTAGAAACAA | 59.5 | chr7:122221094-122221492 | 399bp |
| --- | --- | --- | --- | --- | --- | --- |
|  |  |  | **CADPS2-7R**  GGGCTTGTGGGAAATAACAA | 59.8 |  |  |
|  | 8 | chr7:122194604-122194743 | **CADPS2-8F**  TCAGCTATTGTAGTAGGGAGTCA | 57.9 | chr7:122194496-122194895 | 400bp |
|  |  |  | **CADPS2-8R**  ACTCCATGTGAAAGCCTGCT | 59.6 |  |  |
|  | 9 | chr7:122153303-122153369 | **CADPS2-9F**  TCCCTTTCGAGAACCCAGAA | 58.3 | chr7:122153059-122153564 | 506bp |
|  |  |  | **CADPS2-9R**  GAGGGTAAAGAGTCCAAAAGCA | 58.3 |  |  |
|  | 10 | chr7:122131366-122131474 | **CADPS2-10F**  GCTGACAAACGAAAGGTGCT | 59.3 | chr7:122131185-122131655 | 471bp |
|  |  |  | **CADPS2-10R**  GCTAAAAATACAAGCCCTCCAA | 59.6 |  |  |
|  | 11 | chr7:122130135-122130335 | **CADPS2-11F**  TGGGAGCTTAACCCTTTTTG | 59.2 | chr7:122129942-122130530 | 589bp |
|  |  |  | **CADPS2-11R**  ACCACACGTACCATTTACTCCA | 59.4 |  |  |
|  | 12 | chr7:122120906-122120914 | **CADPS2-12F**  TCCTCCAGAGTTCCTCCAGA | 59.0 | chr7:122120787-122121141 | 355bp |
|  |  |  | **CADPS2-12R**  CTGGGTTTGGCTAGAAAGTAGG | 58.4 |  |  |
|  | 13 | chr7:122114435-122114571 | **CADPS2-13F**  TGCTGAACTCTTGGCATCAT | 57.5 | chr7:122114221-122114712 | 492bp |
|  |  |  | **CADPS2-13R**  CAGCAGTGGTCTTCAAGTGT | 58.0 |  |  |
|  | 14 | chr7:122111429-122111616 | **CADPS2-14F**  ACAAGTTACCCATGCCTTGC | 60.0 | chr7:122111207-122111852 | 646bp |
|  |  |  | **CADPS2-14R**  TCTTTCGAGTGGCTTTACCAG | 59.5 |  |  |

**S3 Table. Continued**

|  | 15 | chr7:122091428-122091529 | **CADPS2-15F**  TCCATTCATTCTGGGTCAAA | 58.9 | chr7:122091256-122091714 | 456bp |
| --- | --- | --- | --- | --- | --- | --- |
|  |  |  | **CADPS2-15R**  TACGCTGAGAAGCAGACCAC | 59.2 |  |  |
|  | 16 | chr7:122081566-122081629 | **CADPS2-16F**  TCGCTTTACTCATGACCCTTGA | 59.4 | chr7:122081384-122081924 | 541bp |
|  |  |  | **CADPS2-16R**  TCAGGGAGAATGAAGTGCTGT | 59.0 |  |  |
|  | 17 | chr7:122078395-122078518 | **CADPS2-17F**  TGCAGACCCATTACATCCCC | 59.5 | chr7:122078195-122078694 | 500bp |
|  |  |  | **CADPS2-17R**  TTCAAGCAGAGTCCCAGGAG | 59.0 |  |  |
|  | 18 | chr7:122076414-122076434 | **CADPS2-18F**  TTTCATCATCTCCCTCTCCCC | 58.6 | chr7:122076264-122076616 | 353bp |
|  |  |  | **CADPS2-18R**  CGTTTCACAGCTATTTACCTCCA | 58.8 |  |  |
|  | 19 | chr7:122056115-122056218 | **CADPS2-19F**  CCTGTCATTGCTTTGTGGGT | 58.7 | chr7:122055939-122056411 | 473bp |
|  |  |  | **CADPS2-19R**  TGTGAATGTGCAATGGGATGT | 58.5 |  |  |
|  | 20 | chr7:122047594-122047750 | **CADPS2-20F**  TTTCATTCCTCAGTGCCAAA | 59.3 | chr7:122047359-122047949 | 591bp |
|  |  |  | **CADPS2-20R**  TCAGTGAAGGTAGGAGGATGC | 59.3 |  |  |
|  | 21 | chr7:122033495-122033636 | **CADPS2-21F**  GTGTCAGACTCTCTCAGAAAACG | 59.0 | chr7:122033145-122033735 | 591bp |
|  |  |  | **CADPS2-21R**  ACCAACGATGACAAATGCCA | 58.4 |  |  |

**S3 Table. Continued**

|  | 22 | chr7:122033250-122033369 | **CADPS2-22F**  GTGTCAGACTCTCTCAGAAAACG | 59.0 | chr7:122033145-122033735 | 591bp |
| --- | --- | --- | --- | --- | --- | --- |
|  |  |  | **CADPS2-22R**  ACCAACGATGACAAATGCCA | 58.4 |  |  |
|  | 23 | chr7:122028637-122028792 | **CADPS2-23F** TCTCCTTGCCCTGCCTTATT | 59.3 | chr7:122028421-122028987 | 567bp |
|  |  |  | **CADPS2-23R** GGCAATAGTTGGGAGACCTG | 59.6 |  |  |
|  | 24 | chr7:122027080-122027227 | **CADPS2-24F**  TGCCACACTGCTTACTCCTT | 59.2 | chr7:122026965-122027354 | 390bp |
|  |  |  | **CADPS2-24R**  TGGTGCTAGAGAACTTGGTCT | 58.4 |  |  |
|  | 25 | chr7:122019422-122019496 | **CADPS2-25F**  AGTGTTGACTGGGTGGATCA | 58.6 | chr7:122019269-122019663 | 395bp |
|  |  |  | **CADPS2-55R**  TTTTTGCTCCCAACTTTTCTG | 59.9 |  |  |
|  | 26 | chr7:122000984-122001067 | **CADPS2-26F**  CCTCAGCTGTCAGTTCATTCA | 58.0 | chr7:122000697-122001194 | 498bp |
|  |  |  | **CADPS2-26R**  TGAAGGTTAGTGCTTGCTTAAGG | 58.9 |  |  |
|  | 27 | chr7:122000842-122000874 | **CADPS2-27F**  CCTCAGCTGTCAGTTCATTCA | 58.0 | chr7:122000697-122001194 | 498bp |
|  |  |  | **CADPS2-27R**  TGAAGGTTAGTGCTTGCTTAAGG | 58.9 |  |  |
|  | 28 | chr7:121985628-121985735 | **CADPS2-28F**  GCTTGACATACTGGCCTTTCA | 60.3 | chr7:121985423-121986022 | 600bp |
|  |  |  | **CADPS2-28R**  CTCAACTGATCCACCGGTCT | 60.1 |  |  |

**S3 Table. Continued**

|  | 29 | chr7:121965531-121965635 | **CADPS2-29F**  ACAGCCTAGGTCAACTTAACAGA | 59.1 | chr7:121965256-121965840 | 585bp |
| --- | --- | --- | --- | --- | --- | --- |
|  |  |  | **CADPS2-29R**  TTGTGAACCTACTTCCTTCAGG | 57.7 |  |  |
|  | 30 | chr7:121958478-121960392 |  |  |  |  |
|  | 30.1 |  | **CADPS2-30.1F**  GGACAGCCACATGAACCTATC | 58.4 | chr7:121959728-121960515 | 788bp |
|  |  |  | **CADPS2-30.1R**  AGTACAAAACGAACCTGGCAC | 59.1 |  |  |
|  | 30.2 |  | **CADPS2-30.2F**  TGTAGAGTAAAAGGGGAAATGCC | 58.4 | chr7:121959211-121960079 | 869bp |
|  |  |  | **CADPS2-30.2R**  CCTCTGCTATGGAAATATGCTGT | 58.4 |  |  |
|  | 30.3 |  | **CADPS2-30.3F**  AGGTGTGGGGTAGCAAGTG | 59.2 | chr7:121958720-121959544 | 825bp |
|  |  |  | **CADPS2-30.3R**  TCCCGGAAGAAGGAAGAAATTT | 57.6 |  |  |
|  | 30.4 |  | **CADPS2-30.4F**  TTTCTTGCTAGGGTTTGAGAGA | 57.0 | chr7:121958297-121959096 | 800bp |
|  |  |  | **CADPS2-30.4R**  CTCCAGGCCGCAAATACTTT | 58.5 |  |  |
|  |  |  |  |  |  |  |
| *STPG1* | 1 | chr1:24740164-24740262 | **STPG1-1F**  GTGTCCTCTTCCCCACGAC | 60.5 | chr1:24740108-24740352 | 245bp |
|  |  |  | **STPG1-1R**  GCTTCTCTGCGGACTTTGAA | 60.7 |  |  |

**S3 Table. Continued**

|  | 2 | chr1:24727809-24727946 | **STPG1-2F**  ACCCAGGACTTACTATTCCCC | 58.3 | chr1:24727717-24728071 | 355bp |
| --- | --- | --- | --- | --- | --- | --- |
|  |  |  | **STPG1-2R**  ACTTGGTAGATCTCAGCCGG | 59.3 |  |  |
|  | 3 | chr1:24718051-24718169 | **STPG1-3F**  CCCTCAGTGGAGCTGGTG | 60.4 | chr1:24717905- 24718298 | 394bp |
|  |  |  | **STPG1-3R**  TCCTGTCAAAAGGCAAGTTTCT | 60.3 |  |  |
|  | 4 | chr1:24710392-24710493 | **STPG1-4F**  TTCCTCTGGCTTCTCTTCCC | 60.8 | chr1:24710166-24710651 | 486bp |
|  |  |  | **STPG1-4R**  AGTCAGGAAATGGTGGAGCT | 58.7 |  |  |
|  | 5 | chr1:24706143-24706313 | **STPG1-5F**  GATGTCCCCAAGATCCCCTC | 62.9 | chr1:24706059-24706439 | 381bp |
|  |  |  | **STPG1-5R**  CTGCCAGAGTTTCGCCTTTT | 61.8 |  |  |
|  | 6 | chr1:24700192-24700300 | **STPG1-6F**  CTGTGAAGAAGTAGGTGCCC | 57.4 | chr1:24700111-24700491 | 381bp |
|  |  |  | **STPG1-6R**  AGACCACTTGAGCTGGGATG | 60.3 |  |  |
|  | 7 | chr1:24696164-24696329 | **STPG1-7F**  GCAGTGACACCCCATCCTTT | 62.3 | chr1:24696078-24696473 | 396bp |
|  |  |  | **STPG1-7R**  GGCTATCTCAGTCAGCCCTT | 58.5 |  |  |
|  | 8 | chr1:24687341-24687531 | **STPG1-8F**  TCTACCCTCATCCCTGTAACC | 58.0 | chr1:24687217-24687595 | 379bp |
|  |  |  | **STPG1-8R**  TAGAAACTGTGCCTTCCCCA | 60.6 |  |  |

**S3 Table. Continued**

|  | 9 | chr1:24683489-24685109 |  |  |  |  |
| --- | --- | --- | --- | --- | --- | --- |
|  | 9.1 |  | **STPG1-9.1F**  GTCCTAATTCCCAAGCCCCT | 61.5 | chr1:24683399-24684051 | 653bp |
|  |  |  | **STPG1-9.1R**  GTTCTCAGCCTTGACTGCAC | 58.6 |  |  |
|  | 9.2 |  | **STPG1-9.2F**  TTCCCACCTCCTGAACTTCC | 61.4 | chr1:24683822-24684796 | 975bp |
|  |  |  | **STPG1-9.2R**  CAGAAGGGAGGAGTGGACAG | 59.8 |  |  |
|  | 9.3 |  | **STPG1-9.3F**  TGAGAAAGGCAGGAGTGAGG | 60.7 | chr1:24684572-24685259 | 688bp |
|  |  |  | **STPG1-9.3R**  GCCAGTTCCTTCCTCTCTGT | 58.5 |  |  |
