# Supplementary material for: Accurate Breakpoint Mapping in Apparently Balanced Translocation Families with Discordant Phenotypes Using Whole Genome Mate-Pair Sequencing
Source: PLoS One. 2017 Jan 10;12(1):e0169935. doi: 10.1371/journal.pone.0169935 (PMC5225008; doi:10.1371/journal.pone.0169935)
Supplement: S4 Table — List of fluorescent chromosome 7-specific repeat microsatellite markers used for UPD analysis in the affected t(1;7)(p36.1;q22) translocation carrier in family 1, non-affected t(1;7)(p36.1;q22) translocation carrier mother and non-affected father. Peak sizes from each marker in each sample are indicated as well as whether the result was informative (I) or not (NI). Based on the informative results, normal biparental inheritance was concluded. Results from D7S1824 (marked with an asterisk) were used as an example in Fig 1C (main manuscript). (DOC) [file pone.0169935.s009.doc]

**S4 Table. Uniparental Disomy 7 (UPD7) results for family 1.**

List of fluorescent chromosome 7-specific repeat microsatellite markers used for UPD analysis in the affected t(1;7)(p36.1;q22) translocation carrier in family 1, non-affected t(1;7)(p36.1;q22) translocation carrier mother and non-affected father. Peak sizes from each marker in each sample are indicated as well as whether the result was informative (I) or not (NI). Based on the informative results, normal biparental inheritance was concluded. Results from D7S1824 (marked with an asterisk) were used as an example in Fig 1C (main manuscript).

| **chr7 marker name** | **Size (bp)** | **Dye** | **Position** | **Patient’s Result** | **Mother’s Result** | **Father’s Result** |  |
| --- | --- | --- | --- | --- | --- | --- | --- |
| D7S2201 | 101-117 | PET  red | 7p22.1 | 109/112 | 112/112 | 109/112 | NI |
| D7S817 | 150-177 |  | 7p14.3 | 171/171 | 171/171 | 167/171 | NI |
| D7S2204 | 217-269 |  | 7q21.11 | 239/251 | 251/255 | 231/239 | I |
| D7S483 | 160-188 | NED  black | 7q36.1 | 175/180 | 175/180 | 178/180 | NI |
| D7S2212 | 192-216 |  | 7q21.11 | 197/197 | 197/197 | 197/197 | NI |
| D7S1808 | 250-276 |  | 7p15.2-p15.1 | 254/270 | 254/266 | 258/270 | I |
| D7S1824 | 163-205 | FAM  blue | 7q34 | 187/195 | 195/199 | 167/187 | I |
| D7S519 | 255-268 |  | 7p12.3 | 257/259 | 257/259 | 257/265 | NI |
