# Supplementary material for: Accurate Breakpoint Mapping in Apparently Balanced Translocation Families with Discordant Phenotypes Using Whole Genome Mate-Pair Sequencing
Source: PLoS One. 2017 Jan 10;12(1):e0169935. doi: 10.1371/journal.pone.0169935 (PMC5225008; doi:10.1371/journal.pone.0169935)
Supplement: S5 Table — chr. = chromosome; F = forward; R = reverse; Tm = melting temperature; bp = base pairs. (DOC) [file pone.0169935.s010.doc]

**S5 Table. qRT-PCR primer sequences used for the validation of selected structural variants.**

chr.=chromosome; F=forward; R=reverse; Tm=melting temperature; bp=base pairs.

| **Structural Variant** | **qRT-PCR primer name** | **qRT-PCR primer sequence** | **Tm (^o^C)** | **Amplified region (hg19)** | **Amplicon size** |
| --- | --- | --- | --- | --- | --- |
|  | Control-F | GGCCCAGGACTTATCTCGAC | 61.0 | chr18:33047856-33047980 | 125bp |
|  | Control-R | GTACAGGATGCCACCCCTCT | 61.3 |  |  |
| *CACNA2D2* duplication identified in the affected translocation carrier in family 1 | C-RT1F | GCATGTTGTGACCATTCCTG | 60.0 | chr3:50388686-50388813 | 128bp |
|  | C-RT1R | ATCTCAGGTGCCCACTGTTC | 60.1 |  |  |
|  | C-RT2F | GTTGCCCTTTTTCCAAGTGA | 60.1 | chr3:50397652-50397784 | 133bp |
|  | C-RT2R | CCTCTCATCCTCCGCAGTTA | 60.3 |  |  |
|  | C-RT3F | TCCTCACTGTCGCGTTGTAG | 60.0 | chr3:50402312-50402392 | 81bp |
|  | C-RT3R | CGGAGCAGTGTGAGCTAGTG | 59.8 |  |  |
|  | C-RT4F | CTCCCCATGGAGTCGTCTTA | 60.0 | chr3:50402993-50403116 | 124bp |
|  | C-RT4R | CCTGTTCCAGCAGCTTCTCT | 59.8 |  |  |
| *ZNF423* deletion identified in the affected translocation carrier in family 2 | Z-RT1F | AAATCTGCTCCTCTGCTCCA | 60.1 | chr16:49746990-49747084 | 95bp |
|  | Z-RT1R | AGCTTTTGGTGGTCCCTTTT | 60.0 |  |  |
|  | Z-RT2F | GCAGAATCTTCCCACCTTGA | 60.2 | chr16:49755778-49755908 | 131bp |
|  | Z-RT2R | AGAGCATCCCTCCCTTGACT | 60.2 |  |  |
|  | Z-RT3F | CTTGTTGCACAGCAGCATTT | 60.1 | chr16:49758653-49758761 | 109bp |
|  | Z-RT3R | CCTCTCCGCTATCCCTTCTT | 59.8 |  |  |
