# Supplementary material for: Accurate Breakpoint Mapping in Apparently Balanced Translocation Families with Discordant Phenotypes Using Whole Genome Mate-Pair Sequencing
Source: PLoS One. 2017 Jan 10;12(1):e0169935. doi: 10.1371/journal.pone.0169935 (PMC5225008; doi:10.1371/journal.pone.0169935)
Supplement: S6 Table — (DOC) [file pone.0169935.s011.doc]

**S6 Table. List of filtered structural variants (SVs) (≥5 reads), not overlapping with any Database of Genomic Variants entry, found uniquely in the affected member of family 1.**

| **SV no.** | **SV Breakpoint Junctions as predicted by MPS (hg19)** | **Predicted SV size** | **Type of SV / number of read-pairs supporting SV** | **Disrupted Gene(s)** |
| --- | --- | --- | --- | --- |
| 1 | chr1:94040257-94045847 | 5,591bp | DELETION_UNBAL_6reads | *BCAR3* - breast cancer anti-estrogen resistance 3 |
| 2 | chr1:182761132-182767271 | 6,140bp | DELETION_UNBAL_5reads | *NPL* - N-acetylneuraminate pyruvate lyase |
| 3 | chr2:130193884-130202391 | 8,508bp | TRANSLOC_BAL_5reads | No gene disrupted |
| 4 | chr2:160095442-160101886 | 6,445bp | INV_FRAGMT_BAL_6reads | *WDSUB1* - WD repeat, sterile alpha motif and U-box domain containing 1 |
| 5 | chr4:190191420-190200668 | 9,249bp | TRANSLOC_BAL_17reads | No gene disrupted |
| 6 | chr5:14663689-14672431 | 8,743bp | TRANSLOC_BAL_5reads | *FAM105B* - *OTULIN* - OTU deubiquitinase with linear linkage specificity |
| 7 | chr6:119539813-119542295 | 2,483bp | INV_FRAGMT_BAL_18reads | *MAN1A1* - mannosidase, alpha, class 1A, member 1 |
| 8 | chr8:32719889-32727327 | 9,080bp | DELETION_UNBAL_5reads | No gene disrupted |
| 9 | chr8:32722475-32728968 | 6,494bp | DELETION_UNBAL_5reads | No gene disrupted |
| 10 | chr8:69870963-69876389 | 5,427bp | DELETION_UNBAL_7reads | *NR_039986* - LINC01592 long intergenic non-protein coding RNA 1592 |
| 11 | chr8:87942833-87948873 | 6,041bp | DELETION_UNBAL_5reads | *CNBD1* - cyclic nucleotide binding domain containing 1 |
| 12 | chr8:102700447-102708327 | 7,881bp | DELETION_UNBAL_5reads | *NCALD1* - Neurocalcin delta |
| 13 | chr10:124839138-124845705 | 6,568bp | DELETION_UNBAL_5reads | No gene disrupted |
| 14 | chr10:127185598-127199073 | 13,476bp | INV_DUPLI_UNBAL_6reads | No gene disrupted |
| 15 | chr16:9964723-9969455 | 4,732bp | TRANSLOC_BAL_5reads | *GRIN2A* - glutamate receptor, ionotropic, N-methyl D-aspartate 2A |
| 16 | chr17:75017471-75024749 | 7,279bp | DELETION_UNBAL_5reads | No gene disrupted |
| 17 | chrX:2832015-2832195 | 181bp | INV_TRANSLOC_BAL_11reads_chrY:14491215-14491429 | *ARSD* - arylsulfatase D |
| 18 | chrY:13212279-13217540 | 5,262bp | TRANSLOC_BAL_5reads | No gene disrupted |
| 19 | chrY:14491215-14491429 | 215bp | INV_TRANSLOC_BAL_11reads_chrX:2832015-2832195 | No gene disrupted |
