# Supplementary material for: Accurate Breakpoint Mapping in Apparently Balanced Translocation Families with Discordant Phenotypes Using Whole Genome Mate-Pair Sequencing
Source: PLoS One. 2017 Jan 10;12(1):e0169935. doi: 10.1371/journal.pone.0169935 (PMC5225008; doi:10.1371/journal.pone.0169935)
Supplement: S7 Table — (DOC) [file pone.0169935.s012.doc]

**S7 Table. List of filtered structural variants (SVs) (≥5 reads), not overlapping with any Database of Genomic Variants** entry, found uniquely in the affected member of family 2.

| **SV no.** | **SV Breakpoint Junctions as predicted by MPS (hg19)** | **Predicted SV size** | **Type of SV / number of read-pairs supporting SV** | **Disrupted Gene(s)** |
| --- | --- | --- | --- | --- |
| 1 | chr2:109875017-109879287 | 4,271bp | INV_FRAGMT_BAL_5reads | *SH3RF3* - SH3 domain containing ring finger 3 |
| 2 | chr6:58771781-58783978 | 12,198bp | INS_FRAGMT_BAL_5reads_ chr7:61970088-61976429 | No gene disrupted |
| 3 | chr7:61970088-61976429 | 6,342bp | INS_FRAGMT_BAL_5reads_ chr6:58771781-58783978 | No gene disrupted |
| 4 | chr11:71278206-71293023 | 14,818bp | INV_DUPLI_UNBAL_13reads | No gene disrupted |
| 5 | chr16:49741265-49760865 | 19,601bp | DELETION_UNBAL_17reads | *ZNF423* - zinc finger protein 423 |
| 6 | chr16:80975114-80984001 | 8,888bp | TRANSLOC_BAL_5reads | No gene disrupted |
