# Supplementary material for: Accurate Breakpoint Mapping in Apparently Balanced Translocation Families with Discordant Phenotypes Using Whole Genome Mate-Pair Sequencing
Source: PLoS One. 2017 Jan 10;12(1):e0169935. doi: 10.1371/journal.pone.0169935 (PMC5225008; doi:10.1371/journal.pone.0169935)
Supplement: S8 Table — (DOC) [file pone.0169935.s013.doc]

**S8 Table. List of filtered structural variants (SVs) (≥5 reads), not overlapping with any Database of Genomic Variants entry, found uniquely in the affected member of family 3.**

| **SV no.** | **SV Breakpoint Junctions as predicted by MPS (hg19)** | **Predicted SV size** | **Type of SV / number of read-pairs supporting SV** | **Disrupted Gene(s)** |
| --- | --- | --- | --- | --- |
| 1 | chr1:63254802-63256431 | 1,630bp | INV_FRAGMT_BAL_4reads | *ATG4C* – autophagy related 4C, cysteine peptidase |
| 2 | chr1:199109769-199113921 | 4,153bp | TRANSLOC_BAL_12reads | No gene disrupted |
| 3 | chr3:98410311-98415001 | 4,691bp | TRANSLOC_BAL_8reads | No gene disrupted |
| 4 | chr4:99250903-99256759 | 5,857bp | DELETION_UNBAL_5reads | *RAP1GDS1* - RAP1, GTP-GDP dissociation stimulator 1 |
| 5 | chr5:121575940-121581043 | 5,104bp | TRANSLOC_BAL_12reads | No gene disrupted |
| 6 | chr5:124988681-124993498 | 4,818bp | DELETION_UNBAL_5reads | No gene disrupted |
| 7 | chr7:19049860-19056263 | 6,404bp | TRANSLOC_BAL_5reads | No gene disrupted |
| 8 | chr10:67306485-67315396 | 8,912bp | TRANSLOC_BAL_6reads | No gene disrupted |
| 9 | chr13:58019841-58029937 | 10,097bp | INS_FRAGMT_BAL_5reads | No gene disrupted |
| 10 | chr16:33241076-33294270 | 53,195bp | TRANSLOC_BAL_5reads | *TP53TG3* - TP53 target 3 |
| 11 | chr19:37731253-37733108 | 1,857bp | INV_FRAGMT_BAL_5reads | *ZNF383* - Zinc finger protein 383 |
