# Supplementary material for: Accurate Breakpoint Mapping in Apparently Balanced Translocation Families with Discordant Phenotypes Using Whole Genome Mate-Pair Sequencing
Source: PLoS One. 2017 Jan 10;12(1):e0169935. doi: 10.1371/journal.pone.0169935 (PMC5225008; doi:10.1371/journal.pone.0169935)
Supplement: S9 Table — (DOC) [file pone.0169935.s014.doc]

**S9 Table. List of filtered structural variants (SVs) (≥5 reads), not overlapping with any Database of Genomic Variants** entry, found uniquely in the affected member of family 4.

| **SV no.** | **SV Breakpoint Junctions as predicted by MPS (hg19)** | **Predicted SV size** | **Type of SV / number of read-pairs supporting SV** | **Disrupted Gene(s)** |
| --- | --- | --- | --- | --- |
| 1 | chr3:68634509-68640367 | 5,859bp | TRANSLOC_BAL_11reads | No gene disrupted |
| 2 | chr5:5682301-5714557 | 32,257bp | TRANSLOC_BAL_11reads | No gene disrupted |
| 3 | chr6:127655806-127755756 | 99,951bp | LARGE_DUPLI_UNBAL_10reads | *ECHDC1* - enoyl CoA hydratase domain containing 1 |
| 4 | chr6:127655820-127681714 | 25,895bp | INVERSION_UNBAL_10reads | *ECHDC1* - enoyl CoA hydratase domain containing 1 |
| 5 | chr12:37989730-37998790 | 9,061bp | TRANSLOC_BAL_7reads | No gene disrupted |
| 6 | chr12:58571913-58574947 | 3,035bp | INV_FRAGMT_BAL_5reads | No gene disrupted |
